# Continuous modeling of creased annuli with tunable bistable and looping behaviors

**DOI:** 10.1073/pnas.2209048120

**Published:** 2023-01-20

**Authors:** Tian Yu, Francesco Marmo, Pasquale Cesarano, Sigrid Adriaenssens

**Affiliations:** ^a^Department of Civil and Environmental Engineering, Princeton University, Princeton, NJ 08544; ^b^Department of Structures for Engineering and Architecture, University of Naples Federico II, 80131, Naples, Italy

**Keywords:** discontinuity, creased annuli, bistability, foldability

## Abstract

Creases are purposely introduced to thin structures for designing deployable origami, artistic geometries, and functional structures with tunable nonlinear mechanics. Modeling the mechanics of creased structures is challenging because creases introduce geometric discontinuity and often have complex mechanical responses due to local material damage. In this work, we propose a continuous description of the sharp geometry of creases and apply it to the study of creased annuli, made by introducing radial creases to annular strips with the creases annealed to behave elastically. We find that creased annuli have generic bistability and can be folded into various compact shapes, depending on the crease pattern and the overcurvature of the flat annulus. We use a regularized Dirac delta function (RDDF) to describe the geometry of a crease, with the finite spike of the RDDF capturing the localized curvature. Together with anisotropic rod theory, we solve the nonlinear mechanics of creased annuli, with its stability determined by the standard conjugate point test. We find excellent agreement between precision tabletop models, numerical predictions from our analytical framework, and modeling results from finite element simulations. We further show that by varying the rest curvature of the thin strip, dynamic switches between different states of creased annuli can be achieved, which could inspire the design of deployable and morphable structures. We believe that our smooth description of discontinuous geometries will benefit the mechanical modeling and design of a wide spectrum of engineering structures that embrace geometric and material discontinuities.

## Significance

Discontinuities are intngeometric properties. Studying the mechanics of these structures often requires cutting the discontinuity and imposing appropriate matching conditions. We propose a robust continuous description of discontinuities in slender structures and successfully apply it to solving the mechanics of creased annuli, a structure with tunable bistable and looping behaviors. We further develop an experimental method capable of constructing precise models of elastic creased annuli, whose mechanical behaviors match well with our numerical modeling results. The continuous framework significantly simplifies the mechanical modeling of creased annuli and could address a large range of material and geometric discontinuities in both 1D slender structures and 2D surfaces.

Abrupt changes are ubiquitous in nature and modern engineering, such as the jump of cross-sections at a tree fork, impact forces, mantle discontinuities (1), the interfaces of shock waves (2), and transient behaviors in signal processing (3), just to name a few. These rapid changes correspond to a step-like jump or a localized spike, which can be described by a Heaviside function and a Dirac delta function, respectively. In mechanical engineering, material and geometric discontinuities have been introduced to elastic structures for various outcomes, such as optimal mechanical properties in stepped beams (4) and remarkable stretchability in flexible electronics (5, 6). Made by decorating thin sheets with certain crease patterns, origami structures bring great potential in achieving compact folding (7–10), target geometries (11–14), and tunable stiffness and stability in metamaterials (15–17). However, it is challenging to study the mechanics of elastic structures with discontinuities (e.g., creases that correspond to *C*^0^ continuity), which normally require cutting the structure and imposing matching conditions at the discontinuous interfaces (18, 19).

In origami structures, creases have been modeled as discrete hinges (18, 20–24), smooth folds with *C*^1^ continuity (25–27), and as thinner (28, 29) or narrower (30) structural elements, which either need a careful specification of matching conditions between the creases and the joining facets or require a detailed definition of the crease region. Recently, Jules et al. used the Heaviside feature of a hyperbolic tangent function to describe the local geometry of creases as *C*^∞^ continuity and studied the mechanics of creased elastica (31).

Here, we propose a Δ function that includes both a boxcar feature and a Dirac delta feature. We use the latter for a continuous description of the crease geometry and implement it with anisotropic rod theory to study the nonlinear mechanics of creased annuli, which are found to have generic bistability and rich looping behaviors. We find excellent agreement between precision tabletop models and numerical predictions from anisotropic rod theory, both showing that the bistability and looping behaviors can be tuned by varying the crease pattern and the overcurvature of the flat annulus. Overall, our work creates opportunities for folding strips into various shapes by introducing creases. The analytical framework could facilitate the mechanics design of thin structures embedded with discontinuities for desired mechanical and geometric properties.

## Δ Function

In this work, we use the spike of a regularized Dirac delta function (RDDF) to model the localized curvature of a crease. We propose the following formulation consisting of two hyperbolic tangent functions,

[1] $$\begin{array}{c} \Delta_{C}^{\left(l_{b},l_{e}\right)}=\frac{1}{2(l_{e}-l_{b})}[\tanh\left(\frac{x-l_{b}}{C}\right)-\tanh\left(\frac{x-l_{e}}{C}\right)]. \end{array}$$

**Fig. 1.**
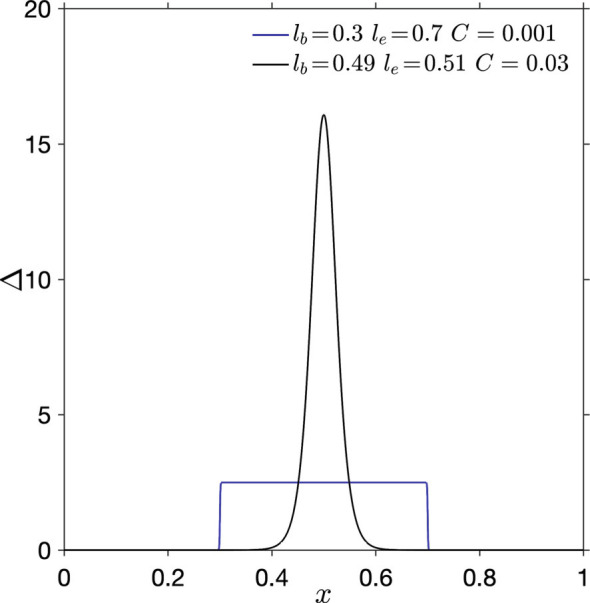
Δ in Eq. **1** can mimic a boxcar profile (blue) and a regularized Dirac delta function (black).

First, we report several properties of Δ that may be used for the continuous description of discontinuities. When *C*​ ≪ ​(*l*_*e*_ − *l*_*b*_), the two steps of hyperbolic tangents are separated, and Δ appears to be a boxcar function, with its value being a constant 1/(*l*_*e*_ − *l*_*b*_) in the range *x* ∈ [*l*_*b*_, *l*_*e*_] and zero elsewhere (blue curve in Fig. 1). As *C* → 0, Δ approaches a perfect boxcar function. When (*l*_*e*_ − *l*_*b*_)< *C* ∪ (*l*_*e*_ − *l*_*b*_)∼*C*, the two steps of hyperbolic tangents collide, and Δ mimics a regularized Dirac delta function (RDDF), symmetric about and maximized at *x* = (*l*_*e*_ + *l*_*b*_)/2 (Black curve in Fig. 1). As (*l*_*e*_ − *l*_*b*_)→0 and *C* → 0, Δ approaches the Dirac delta function, with its value being infinite at *x* = *l*_*b*_ = *l*_*e*_. In addition, it can be shown that ∫_−∞_^∞^Δ*dx* = 1 (*SI Appendix*, section 1). When we use Δ to model the localized curvature of creases, this plays an important role in obtaining target crease angles, which are determined by the integral of Δ.

The above properties of Δ offer promise for describing various types of discontinuities. For example, using the boxcar feature of Δ, we can describe any piecewise continuous function as a single continuous piece with *C*^∞^ (*SI Appendix*, section 2 reports an example characterizing the New York City skyline). In the rest of the paper, we use Δ as an RDDF to model the curvature of creases in elastic strips and investigate the nonlinear mechanics of creased annuli.

## Continuous Description of Creases/Kinks in Anisotropic Rods

**Fig. 2.**
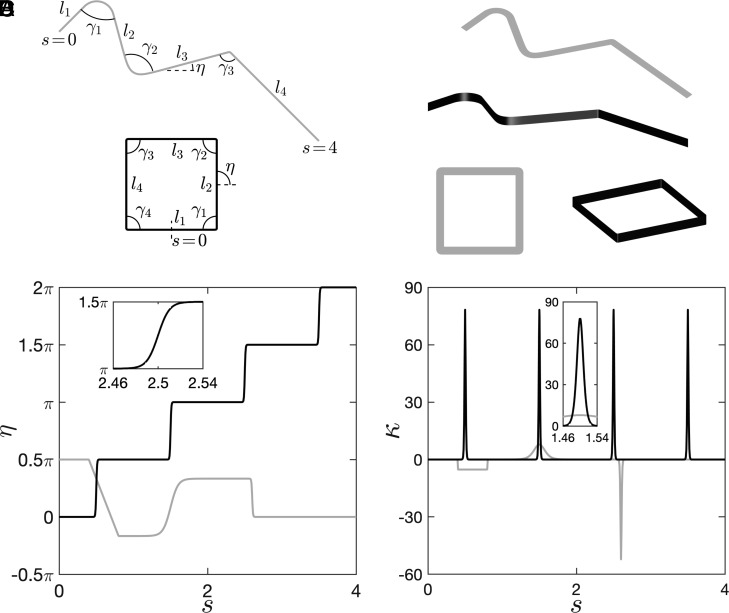
Strips with creases/kinks with the rest curvature described by Eqs. **4** and **2**. (*A*) Rods with multiple creases. The accordion has *n*_*c*_ = 3, (*γ*_1_, *C*_1_, *l*_*b*1_, *l*_*e*1_)=(*π*/3, 0.001, 0.4, 0.8), (*γ*_2_, *C*_2_, *l*_*b*2_, *l*_*e*2_)=(*π*/2, 0.1, 1.499, 1.501), and (*γ*_3_, *C*_3_, *l*_*b*3_, *l*_*e*3_)=(2*π*/3, 0.01, 2.599, 2.601). The square has *n* = 4 and (*γ*_*i*_, *C*_*i*_, *l*_*bi*_, *l*_*ei*_)=(*π*/2, 0.01, *i* − 0.501, *i* − 0.499). (*B*) The planar rods in (*A*) are used as centerlines to construct strips with kinks (gray) and creases (black). (*C*–*D*) report the tangent angle *η* and the curvature *κ* of the rods in (*A*), respectively.

Ideal creases/kinks have a vanishing extension and correspond to *C*^0^ continuity, i.e., the local tangent is discontinuous and results in a blow-up of the local curvature. Due to the material thickness, creases in thin elastic structures always have a small extension, which results in the localization of the curvature. Here, we combine a series of Δ functions to describe the rest curvature of anisotropic rods with multiple creases/kinks,

[2] $$\begin{array}{c} \begin{array}{cc} \kappa & =\sum_{i=1}^{n_{c}}\;{\text{sgn}}_{i}\frac{\pi -\gamma_{i}}{2(l_{\mathrm{ei}}-l_{\mathrm{bi}})}\\ & \;\times [\tanh\left(\frac{s-l_{\mathrm{bi}}}{C_{i}}\right)-\tanh\left(\frac{s-l_{\mathrm{ei}}}{C_{i}}\right)]\\ & =\sum_{i=1}^{n_{c}}{\left(-1\right)}^{i}\left(\pi -\gamma_{i}\right)\Delta_{C_{i}}^{\left(l_{\mathrm{bi}},l_{\mathrm{ei}}\right)}, \end{array} \end{array}$$

where *n*_*c*_, *γ*_*i*_, *s* (∈[0, *l*]), and *l* represent the number of creases, the *i*_th_ crease angle (Fig. 2*A* for the definition of crease angle), the arc length of the rod, and its total length, respectively. The prefactor sgn_*i*_ could be ±1 and is used to prescribe the bending direction of the crease, e.g., the gray accordion in Fig. 2*A*. *C*_*i*_ and (*l*_*ei*_ − *l*_*bi*_) determine the local crease profile centered at *s*​ = ​(*l*_*ei*_ + *l*_*bi*_)/2. With (*l*_*ei*_ − *l*_*bi*_)​ → ​0, our approach degenerates to Jules et al.’s method that uses a hyperbolic tangent to describe the local tangent angle of creases (31) (*SI Appendix*, section 2). With *C*_*i*_​ ≪ ​(*l*_*ei*_ − *l*_*bi*_), Eq. **2** generates uniform bends in the regions *s* ∈ [*l*_*bi*_, *l*_*ei*_] with constant curvatures.

We define (4*C*_*i*_ + *l*_*ei*_ − *l*_*bi*_) as the nominal crease length, which results in a crease region *s* ∈ [*l*_*bi*_ − 2*C*_*i*_, *l*_*ei*_ + 2*C*_*i*_] and in which most of the crease angle *γ*_*i*_ is achieved. Decreasing *C*_*i*_ and (*l*_*ei*_ − *l*_*bi*_) will sharpen the creases and make them independent of other creases. With the increase of (4*C*_*i*_ + *l*_*ei*_ − *l*_*bi*_), adjacent creases will overlap and generate nonflat facets. As long as (4*C*_*i*_ + *l*_*ei*_ − *l*_*bi*_) is small compared with the length of the adjacent facets, the multiple creases represented by Eq. **2** do not affect each other and behave independently. A comprehensive discussion about the influences of *C*_*i*_ and (*l*_*ei*_ − *l*_*bi*_) on the crease profile and the errors of crease angles are included in *SI Appendix*, section 1.

The geometry of the rod is obtained by first integrating *κ* to get the local tangent angle *η* and then integrating the kinematic equations to obtain the rod profile. Fig. 2*A* displays two examples with the rod normalized to the same length 4: a gray accordion with three unevenly distributed creases of different profiles and a black square with four evenly distributed creases of the same profile. *η* measures the local tangent angle. The gray accordion contains a uniform crease with an angle *γ*_1_ and two nonuniform creases of different profiles. The two examples in Fig. 2*A* are used as centerlines to construct strips with kinks (the width of the strip is coplanar to the centerline) and creases (the width of the strip is perpendicular to the centerline), as shown in Fig. 2*B*.

Fig. 2 *C* and *D* present the distribution of the tangent angle *η* and the rest curvature *κ* of the two configurations in Fig. 2*A*, respectively. At a sharp crease, *η* approaches a jump resulting in a spike in *κ*. Increasing the value of (4*C*_*i*_ + *l*_*ei*_ − *l*_*bi*_) leads to blunter creases with the jump of *η* and the spike of *κ* being smoothed in the horizontal direction. In the remainder of this article, we apply the above framework to study the bistable and looping behaviors of creased annuli.

## Geometry of Creased Annuli and Fabrication of Tabletop Models

**Fig. 3.**
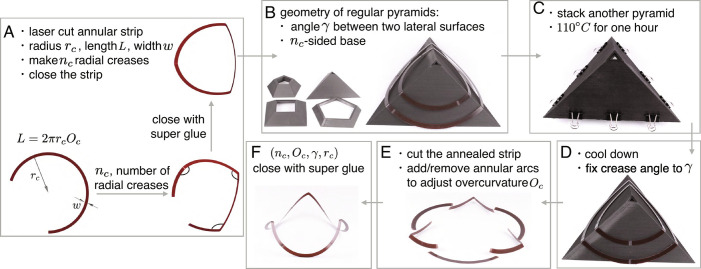
Geometry and fabrication of creased annuli. (*A*) A creased annulus is determined by four geometric parameters (*n*_*c*_, *O*_*c*_, *γ*, *r*_*c*_). (*B*) 3D-printed pyramids that match with the geometry of creased annuli. (*C* and *D*) Annealing. (*E*) Adjust overcurvature. (*F*) Creased annuli with target geometric parameters.

Creased annuli are made by first introducing evenly distributed radial creases of angle *γ* to annular strips (with length *L* and radius of the centerline *r*_*c*_) and then forcing the two ends to close (Fig. 3*A*). We define overcurvature as *O*_*c*_ = *L*/(2*πr*_*c*_), which measures the number of loops the annuli cover in its flat rest state. The geometric parameters of the annular strips include the number of creases *n*_*c*_, overcurvature *O*_*c*_, crease angle *γ*, and the radius *r*_*c*_.

The fabrication process is summarized in Fig. 3. We first laser-cut annular strips with thickness *t*​ = ​0.254 mm, width *w*​ = ​5.08 mm, and length *L* from polyester shim stock (Artus Corp., Englewood, NJ), make *n*_*c*_ radial creases at the desired locations that have been slightly scored by the laser, and close the strip with super glue (Fig. 3*A*). Then, we place the closed strip between two 3D printed pyramids with specific geometries (32), such that the thin strip closely matches the surfaces and ridges of the pyramids (Fig. 3 *B* and *C*). Next, to anneal the strip, we put the pair of stacked pyramids into an oven at 110°*C* for 1 h and cool the sample down at room temperature (≈21 °*C*), which fixes the crease angle to be the ridge angle of the pyramids (i.e., the dihedral angle between two lateral surfaces), eliminates the residual stress inside the crease, and leads to purely elastic creases (31, 33, 34) (Fig. 3 *C* and *D*). After annealing, we cut the strip and insert (remove) additional (redundant) flat annular strips to adjust the overcurvature (Fig. 3*E*). Finally, the structure is closed with super glue to obtain creased annuli with target geometry (*n*_*c*_, *O*_*c*_, *γ*, *r*_*c*_) (Fig. 3*F*). Details of the geometry of the pyramids and the fabrication process are documented in *SI Appendix*, section 3. We find that creased annuli have generic bistability with small overcurvature (*SI Appendix*, Video 1) and can be folded into various shapes by increasing the overcurvature (*SI Appendix*, Video 2).

## Implementation with Anisotropic Rod Theory

Anisotropic rod theory is normally used to model slender rods or strips with a mild anisotropy of the cross-section (35) (i.e. *L* >  > *w* ∼ *t*). Several recent studies have demonstrated its accuracy in predicting the nonlinear mechanics of strips with *w*/*t* up to *O*(10) (36–38). Throughout this study, we fix *w*/*t* to 20 and use the anisotropic rod model to study creased annuli. The force and moment balances of an inextensible and unshearable rod can be summarized as

[3] $$\begin{array}{c} N^{\prime }=0,M^{\prime }+d_{3}\times N=0, \end{array}$$

where a prime denotes an *s* derivative (*s* (∈[0, *l*])), and ***N*** and ***M*** represent contact forces and moments, respectively. ***d***_**3**_ corresponds to the unit tangent vector on the centerline of the strip. We assume linear constitutive relations *M*_1_ = *EI*_1_(*κ*_1_ − *κ*_10_), *M*_2_ = *EI*_2_(*κ*_2_ − *κ*_20_), and *M*_3_ = *GJτ*, where the contact moment *M* has been resolved on a local material frame (*SI Appendix*, section 4). *E* and *G* are Young’s modulus and shear modulus of the constituent isotropic material, respectively; *EI*_1_ and *EI*_2_, and *GJ* correspond to the two bending rigidities and the torsional rigidity, respectively. *κ*_1_, *κ*_2_, and *τ* are the curvatures and twist in the deformed configuration; *κ*_10_ and *κ*_20_ represent the rest curvatures, which correspond to the geodesic curvature of the annulus and the localized curvature of the creases, respectively. Here, we have *κ*_10_ = 2*πO*_*c*_/*L* and $\kappa_{20}=\sum_{i=1}^{n_{c}}\left(\pi -\gamma_{i}\right)\Delta_{C_{i}}^{\left(l_{\mathrm{bi}},l_{\mathrm{ei}}\right)}$. In addition, the length of the strip *l* in numerics is normalized to *n*_*c*_.

To model the sharp creases of creased annuli, we fix (*l*_*ei*_ − *l*_*bi*_) to a small value 2 × 10^−4^*C*_*i*_, which results in a nominal crease length 4*C*_*i*_ (notice that (*l*_*ei*_ − *l*_*bi*_)<  <  *C*_*i*_). In tabletop models, the crease length mainly depends on the thickness *t* of the material and has been shown to be in the order *O*(*t*)∼*O*(10*t*) (31, 39). In order to make the sharpness of the crease in the numerical modeling realistic compared with experimental models, we set 10*t*/(2*πr*_*c*_*O*_*c*_)=4*C*_*i*_/*l*, where we have estimated the crease length to be 10*t* and set the ratio between the crease length and the total strip length to be the same for experiments and numerics. For all the tabletop models, the corresponding *C*_*i*_ is found to be in [8.42 × 10^−4^, 1.03 × 10^−2^].

Notice that we have only set the length of the crease in numerics to be approximately the same as the crease length in experiments; our continuous description of the crease geometry through the specification of *κ*_20_ does not necessarily match with the local crease profile in experiments, which could depend on the material and the creasing method. In *SI Appendix*, section 5, we show that with sharp creases, which are typical for creased thin sheets, the effects of the local crease geometry on the numerical results are negligible. After nondimensionalization, the only material parameter is Poisson’s ratio *ν*, which we set to 0.33 for the current study. Details of the anisotropic rod model, its implementation with numerical continuation package AUTO 07P (40) for solving static equilibria, and the stability test of the equilibria are discussed in *SI Appendix*, section 4. Numerical continuation is powerful for conducting parametric studies and can trace the solutions as a bifurcation parameter varies. AUTO 07P is able to detect various kinds of bifurcations and folds and further compute the bifurcated branches (40).

## Creased Annuli with Tunable Bistability and Looping Behaviors

**Fig. 4.**
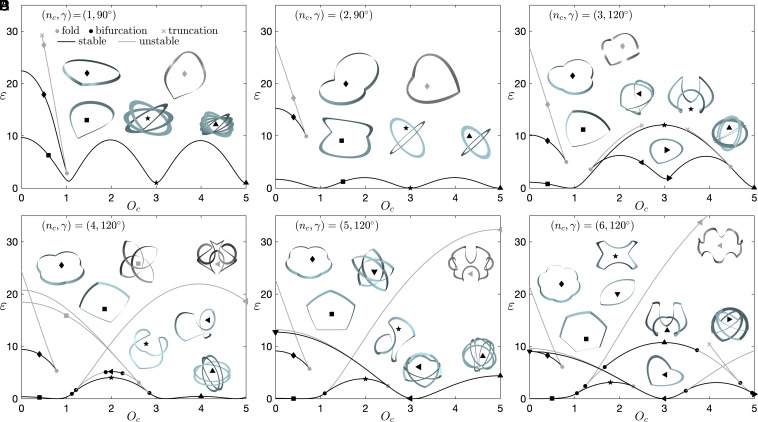
Comparison of experimental models of creased annuli (brown), stable states from numerical predictions of anisotropic rod (blue), and equilibria obtained from finite element simulations (green), with different geometric parameters (*n*_*c*_, *O*_*c*_, *γ*, *r*_*c*_). ()_1, 2_ correspond to a bistable pair. Generic contact is observed in looped states and is not included in numerical modelings.

Comparisons between the numerical results (blue renderings) and experimental models (brown) are summarized in Fig. 4 with different geometric parameters (*n*_*c*_, *O*_*c*_, *γ*, *r*_*c*_). We find excellent agreement, except for the looped configurations where contact exists in physical models and is not included in numerical predictions. The blue renderings are constructed from the anisotropic rod model and have the same slenderness (i.e., *w*/*L*) as the corresponding experimental models. We additionally conduct finite element (FE) simulations using the commercial software ABAQUS, with the modeling results presented in Fig. 4 as green shapes. Creases and overcurvature of creased annuli are generated by applying temperature gradients along both the thickness and width of the cross-section (*Materials and Methods*). All the FE results agree well with our numerical predictions from anisotropic rod theory. This confirms the accuracy of our framework and further implies that self-contact could be the main cause of the differences between the experimental models and the numerical results for the looped configurations. Parametric studies with ABAQUS show that material properties (i.e., Young’s modulus and Poisson’s ratio) have negligible effects on the static equilibria of creased annuli. These findings agree with the conclusions drawn from our theoretical framework (*SI Appendix*, section 4).

We find that creased annuli have generic bistability with small overcurvatures and tunable looping behaviors with large overcurvatures. In the first row of Fig. 4 (*O*_*c*_​ = ​0.7), ()_1_ and ()_2_ represent a bistable pair and are referred to as folded and inverted state, respectively. For example, (*a*)_1_ and (*a*)_2_ are a bistable pair that can be manually deformed to one or the other (*SI Appendix*, Video I). In the third row of Fig. 4 with a large overcurvature *O*_*c*_​ = ​3, creased annuli fold into multiply covered loops, e.g., in (c), (f), (*i*)_2_, (l), and (o). Notice that with three creases, the looped configuration in (*i*)_2_ resembles a triply covered version of (*a*)_1_ and could further be deployed to a stable flower-like shape in (*i*)_1_. With an intermediate overcurvature around 1.5 (the second row in Fig. 4), creased annuli with one, two, there, and four creases are monostable, corresponding to (*b*), (*e*), (*h*), and (*k*), respectively; the creased annulus with five creases is bistable, which can be folded into a star configuration (*n*)_2_. It is known that annular strips without creases will fold into multiply covered loops at an overcurvature of odd integers (i.e., *O*_*c*_ = 3, 5, 7...) (41, 42). Our results show that by introducing radial creases, the out-of-plane mechanical behaviors of annular strips could be significantly enriched, creating various folding patterns and stable configurations that could be tuned by the number of creases and overcurvatures.

**Fig. 5.**
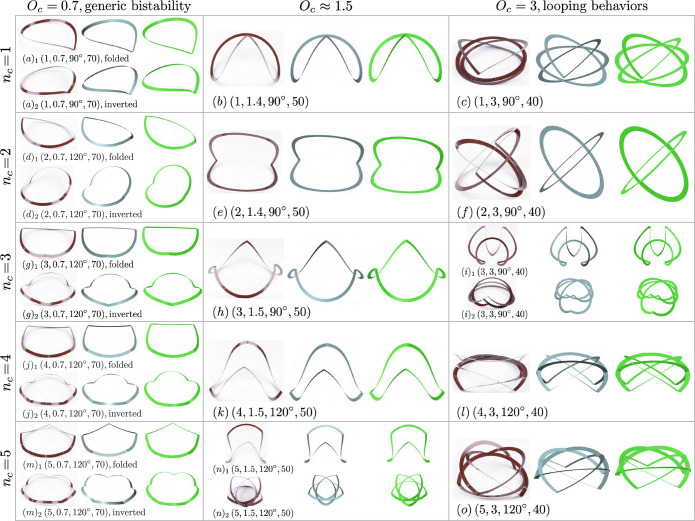
Bifurcation diagrams of creased annuli with fixed crease angle *γ* and different numbers of creases *n*_*c*_. Black and gray curves correspond to stable and unstable solutions, respectively.

We further investigate how overcurvature affects the nonlinear mechanics of creased annuli through numerical continuation. Fig. 5 reports the bifurcation diagram of creased annuli with different numbers of creases in the *O*_*c*_ − *ε* plane, with *ε*​ = ​0.5∫_0_^*l*^[*a*(*κ*_1_ − *κ*_10_)^2^ + *b*(*κ*_2_ − *κ*_20_)^2^ + *τ*^2^] *ds* corresponding to the normalized elastic energy and *a* and *b* being the two normalized bending stiffnesses (*SI Appendix*, section 4). Here, we have fixed (*C*_*i*_, *l*_*ei*_ − *l*_*bi*_) to (0.002, 2 × 10^−4^*C*_*i*_). Black and gray solid circles represent bifurcation and fold points, respectively. Some of the unstable branches are partially reported here and truncated by a gray cross. Renderings represent solutions marked on the curves. Gray and black curves correspond to unstable and stable solutions, respectively. The nonlinear stability of equilibria is obtained by conducting the conventional conjugate point test for a single rod (*SI Appendix*, section 4).

The inverted branch (marked by ⬧) loses stability through a fold point with *O*_*c*_ <  1 for *n*_*c*_ = 2, 3, 4 and 5 and *O*_*c*_​ ≈ ​1 for *n*_*c*_ = 1. On the other hand, with the increase of the overcurvature, the folded shape ■ could directly lead to looped states for *n*_*c*_ = 1, 2 and 3 following the ■​ → ​​⋆​​ → ​​▲ branch or lose stability through bifurcations for *n*_*c*_ = 4, 5 and 6 following the ■​​ → ​​●​​ → ​​⋆ branch, with the bifurcated branch ⋆ connected to looped configuration for *n*_*c*_​ = ​4 or losing stability through a fold point for *n*_*c*_​ = ​5 and 6. In the latter, looped states are independent branches that are disconnected from the folded state ■. For example, the ▼​​ → ​​⊲​​ → ​​▲ branch for *n*_*c*_​ = ​5 and the  ▼​​ → ​​⊲​​ → ​​● branch for *n*_*c*_​ = ​6 represent multiply covered branches. Stable states could also exist in a stability island, for example, the  ⊲​ → ​⊲ branch for *n*_*c*_​ = ​3 (bounded by two fold points), the ⊲ branch for *n*_*c*_​ = ​4 (bounded by two bifurcations), the  ▲ branch for *n*_*c*_​ = ​6 (bounded by two bifurcations), and the ⊲ branch for *n*_*c*_​ = ​6 that gains stability through a bifurcation. Generally speaking, increasing the overcurvature *O*_*c*_ will fold creased annuli into different multiply covered shapes, depending on the number of creases. The looped states normally contain less elastic energy *ε*. In addition, for *n*_*c*_ ≥ 3, increasing *O*_*c*_ deforms the folded branch (■) into a flower-like shape, which is stable for *n*_*c*_ = 3 (⋆) and unstable for *n*_*c*_ = 4, 5-pagination and 6 (⊲).

## Switches Between Different States

Applications of the bistable and looping behaviors in creased annuli may require remote switches between different states. For a thin strip, it is much easier to bend it about the width direction than about the surface normal. Here, we show that adding an actuation curvature $\sum_{i=1}^{n_{a}}\kappa_{20a}\Delta_{C}^{\left(l_{\mathrm{bi}},\;l_{\mathrm{ei}}\right)}$ in the direction of the minimum bending stiffness to *κ*_20_ could trigger dynamic snappings between different states. We adopt the boxcar feature of the Δ function by setting *C* <  < (*l*_*ei*_ − *l*_*bi*_); *n*_*a*_ represents the number of actuated segments and *l*_*bi*_ and *l*_*ei*_ represent the beginning and the end of the *i*_th_ actuated segment, respectively. This allows us to freely vary the number, length, and location of the actuated segments.

**Fig. 6.**
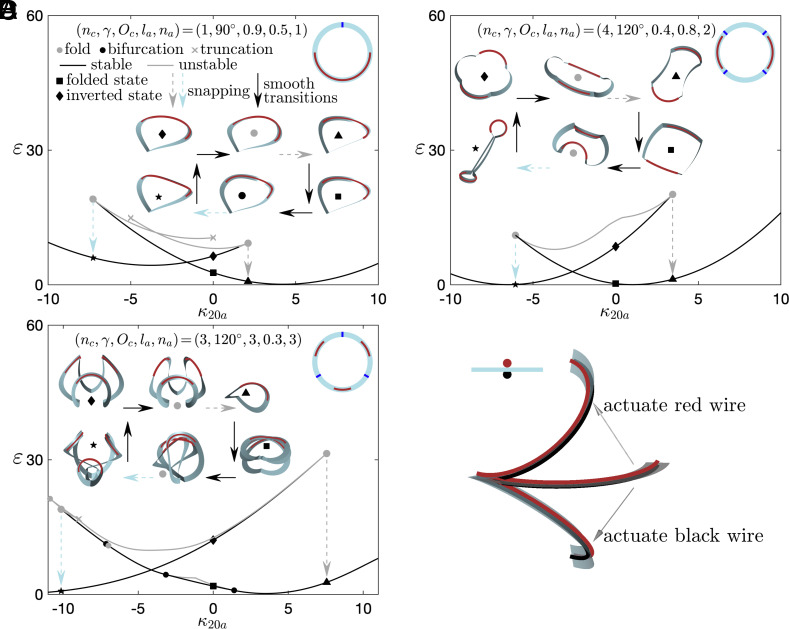
State switches between different states of creased annuli are achieved by introducing an actuation curvature $\sum_{i=1}^{n_{a}}\kappa_{20a}\Delta_{C}^{\left(l_{\mathrm{bi}},l_{\mathrm{ei}}\right)}$ in the direction of the minimum bending stiffness of thin strips. (*A*–*C*): Bifurcation diagrams illustrating state transitions. The blue and red lines in the layout at the top right of each figure represent creases and actuated segments, respectively. (*D*) Schematics of SMA actuation method. Actuating smart memory alloy wires attached to the strip’s upper and bottom surfaces will bend the strip, which appears to introduce the actuation curvature. The inset shows the cross-section of the strip.

Fig. 6*A*–*C* display several bifurcation diagrams under the application of actuation curvatures. Each figure contains a layout at the top right showing creases (blue lines) and actuated segments (red lines), which have the same length *l*_*a*_ (=*l*_*bi*_ − *l*_*ei*_) and are placed symmetrically between adjacent creases. The vertical axis *ε* reports the normalized elastic energy in the structure, namely $0.5\int_{0}^{l}\left[a{\left(\kappa_{1}-\kappa_{10}\right)}^{2}+b{\left(\kappa_{2}-\kappa_{20}-\sum_{i=1}^{n_{a}}\kappa_{20a}\Delta_{C}^{\left(l_{\mathrm{bi}},\;l_{\mathrm{ei}}\right)}\right)}^{2}+\tau^{2}\right]$ *ds*. The solution curves are similar in all three examples: the structure loses stability through a fold point with the increase of |*κ*_20*a*_|, followed by a dynamic jump to a stable state with a lower energy level. Fig. 6*A* shows the transition between the inverted and the folded state of a creased annulus with a single crease. If we start with the inverted state ⬧ and apply a positive *κ*_20*a*_ to half of the strip, the structure loses stability at a fold point and jumps to the folded branch ▲. With the deactivation of *κ*_20*a*_, the structure follows the folded branch to reach the folded state ■. On the other hand, if we start with the folded state ■ and apply a negative *κ*_20*a*_, the structure loses stability through another fold point and snaps back the inverted branch ⋆. With the deactivation of *κ*_20*a*_, the structure follows the inverted branch to reach the inverted state ⬧. This actuation sequence can be applied repeatedly to produce cyclic state switches between the inverted and the folded state. Fig. 6*B* demonstrates similar transitions for a creased annulus with four creases by partially actuating two segments. Fig. 6*C* reports the looping and deployment processes of a creased annulus with three creases, achieved by actuating three short segments.

Notice that there could be many actuation options for achieving transitions between different states of creased annuli. We provide a robust framework that is convenient for designing and further optimizing the actuation scheme because the boxcar feature of the Δ function enables us to freely vary the number, length, and location of the actuated segments. In engineering applications, the actuation curvature can be realized by activating smart memory alloy (SMA) wires attached to the strip surface. When activated, the SMA wire shortens its length and causes the strip to bend in the direction of the minimum bending stiffness due to the eccentricity between the wire’s and the strip’s centerline (Fig. 6*D*). Magnetic actuation provides another solution for generating actuation curvatures by introducing magnetic polarities to the actuated segments (43). The usage of these actuation elements will require the inclusion of the elastic energy of the SMA wire or the magnetic potential, for establishing a full mechanics model.

## Discussion

Through precision tabletop models and numerical predictions from anisotropic rod theory, we demonstrate that introducing radial creases to annular strips enriches their nonlinear mechanics by creating generic bistability and various folding patterns, depending on geometric parameters such as the overcurvature and the crease pattern. Folding of a large closed structure into smaller multiply covered loops is common in slender structures, such as rods/strips (41, 42, 44, 45), curved folding (18), elastic rod networks (46), and ring origami (47). Different from previous results, we have demonstrated tunable looping patterns in creased annuli. Our numerical modeling is based on a continuous description of creases through a regularized Dirac delta function Δ, which captures the localized curvature at creases. We further show that by adding a rest curvature in the direction of the minimum bending stiffness of thin strips, dynamic switches between different states of creased annuli can be achieved. Our framework is convenient for designing and further optimizing the actuation scheme.

In *SI Appendix*, section 5, we show that as long as creases are sharp (i.e., the extension of the crease < < the length of its adjacent facets), any RDDF could be used to study creased annuli without causing notable differences to its nonlinear mechanics. The Δ function proposed in this work could be used to describe other types of material and geometric discontinuities, such as the jump of cross-sections in stepped beams (4) and the jump of rest curvatures in serpentine strips (5). An example of applying our framework to address discontinuous cross-sections and nonlinear material behaviors in the formation of creases is included in *SI Appendix*, section 6. In *SI Appendix*, section 7, we further show that a regularized Heaviside function can describe the geometry of 2D surfaces with discontinuities, which provides opportunities for facilitating the mechanics modeling and design of metasheets (48, 49).

## Materials and Methods

### Fabrication of Elastic Creased Annuli.

To obtain elastic creased annuli with target geometric parameters, we first make stress-free creased annuli through annealing, with its geometry prescribed by the surface of regular pyramids. Then, we cut the annealed annuli and insert/remove flat annular arcs to adjust overcurvature. Based on trial and error, we choose appropriate radii *r*_*c*_ for the tabletop models such that they are not too large and do not suffer significantly from gravity; at the same time, they are not too small and do not cause apparent plastic deformations to the materials. The relation between the geometry of the pyramids and the geometry of creased annuli is detailed in *SI Appendix*, section 3.

### Numerical Implementation in Continuation Package AUTO 07P.

Together with anisotropic rod theory, our continuous modeling of creased annuli is implemented as a two-point boundary value problem in AUTO 07P to conduct numerical continuation. We use a uniform mesh setting in AUTO to make sure that crease regions have enough meshes. The start solution, boundary conditions, and continuation steps are detailed in *SI Appendix*, section 4.

### Nonlinear Stability of Creased Annuli.

Thanks to our description of creased annuli as a single continuous piece, we are able to determine the stability of equilibria through the standard conjugate point test. After obtaining equilibria through numerical continuation, we first solve an initial value problem with the forces and moments being the initial value at *s* = 0; then, stability is determined by examining whether conjugate points exist in the interval *s* ∈ (0, *l*]. Details are documented in *SI Appendix*, section 4.

### Finite Element Modeling.

Finite element analysis is conducted in the commercial software ABAQUS/Standard, and the results are validated against the predictions from our theoretical framework. We choose Timoshenko beams and set the crease length to 10*t*, matching with the crease size implemented in our theoretical framework. The feature “Nlgeom” in ABAQUS is turned on to account for geometric nonlinearity. Temperature gradients are applied along the thickness (only in the crease regions) and the width of the cross-section to introduce the crease angle and the overcurvature to the strip, respectively. Young’s modulus and Poisson’s ratio are set to 1000 MPa and 0.33, respectively.

## Supplementary Material

Appendix 01 (PDF)

Movie S1. **Generic bistability of creased annuli with small overcurvature**. This movie demonstrates generic bistability in creased annuli with Oc = 0.7. The four geometric parameters (*n_c_*, *O_c_*, γ, *r_c_*) correspond to the number of creases, overcurvature of the flat annuli, crease angle, and the radius of curvature of the flat annuli, respectively.

Movie S2. **Looping behaviors of creased annuli with large overcurvature**. This movie demonstrates looping behaviors of creased annuli with large overcurvature. The four geometric parameters (*n_c_*, *O_c_*, γ, *r_c_*) correspond to the number of creases, overcurvature of the flat annuli, crease angle, and the radius of curvature of the flat annuli, respectively. The model with five creases can be folded into a star configuration and the model with three creases can be folded into three loops, with each loop containing one crease.

## Data Availability

All study data are included in the article and/or *SI Appendix*.
