# Supplementary material for: Continuous modeling of creased annuli with tunable bistable and looping behaviors
Source: Proc Natl Acad Sci U S A. 2023 Jan 20;120(4):e2209048120. doi: 10.1073/pnas.2209048120 (PMC9942846; doi:10.1073/pnas.2209048120)
Supplement: Supplementary file 1 — Appendix 01 (PDF) [file pnas.2209048120.sapp.pdf]

Supplementary Information for

Continuous modeling of creased annuli with tunable bistable and looping behaviors

T. Yu, F. Marmo, P. Cesarano, and S. Adriaenssens

Tian Yu
E-mail: tiany@princeton.edu

This PDF file includes:

Supplementary text
Figs. S1 to S10
Tables S1 to S2
Legends for Movies S1 to S2
SI References

Other supplementary materials for this manuscript include the following:

Movies S1 to S2

Supporting Information Text

1. Crease profiles and errors of the crease angle

The Δ function used to describe the rest curvature of creased strips leads to creases with certain profiles and errors of the crease angle. Here, we explore the possible crease profiles and discuss the resulting errors. We focus on a single crease whose rest curvature can be described as,

$$\kappa_0 = \frac{\pi - \gamma}{2(l_e - l_b)}\left[\tanh\left(\frac{s - l_b}{C}\right) - \tanh\left(\frac{s - l_e}{C}\right)\right] = (\pi - \gamma)\Delta_C^{(l_b,l_e)}, \quad [1]$$

with γ corresponding to the target crease angle. $(4C + l_e - l_b)$ and $[l_b - 2C, l_e + 2C]$ represent the nominal crease length and crease region, respectively. We will show that most of the crease angle is formed in the crease region. First, the total area below the Δ function is always unity

$$\int_{-\infty}^{\infty} \Delta_C^{(l_b,l_e)} ds = \frac{C}{2(l_e - l_b)}\left[\ln\left(\cosh\left(\frac{s - l_b}{C}\right)\right) - \ln\left(\cosh\left(\frac{s - l_e}{C}\right)\right)\right]\Big|_{-\infty}^{\infty} = 1. \quad [2]$$

If the rod is infinitely long, we can use Eq. (1) to achieve an exact crease angle γ. However, the length of a realistic rod is always finite, and thus Eq. (1) leads to a crease with the crease angle always bigger than the target angle γ, because the total turning angle of the tangent is always smaller than the desired angle $(\pi - \gamma)$. In addition, with $(l_e - l_b) \to 0$, $\Delta_C^{(l_b,l_e)}$ approaches $\frac{1}{2C\cosh^2\left(\frac{s-l_b}{C}\right)}$, which corresponds to Jules et al.'s description of a single crease in the *elastica* (1).

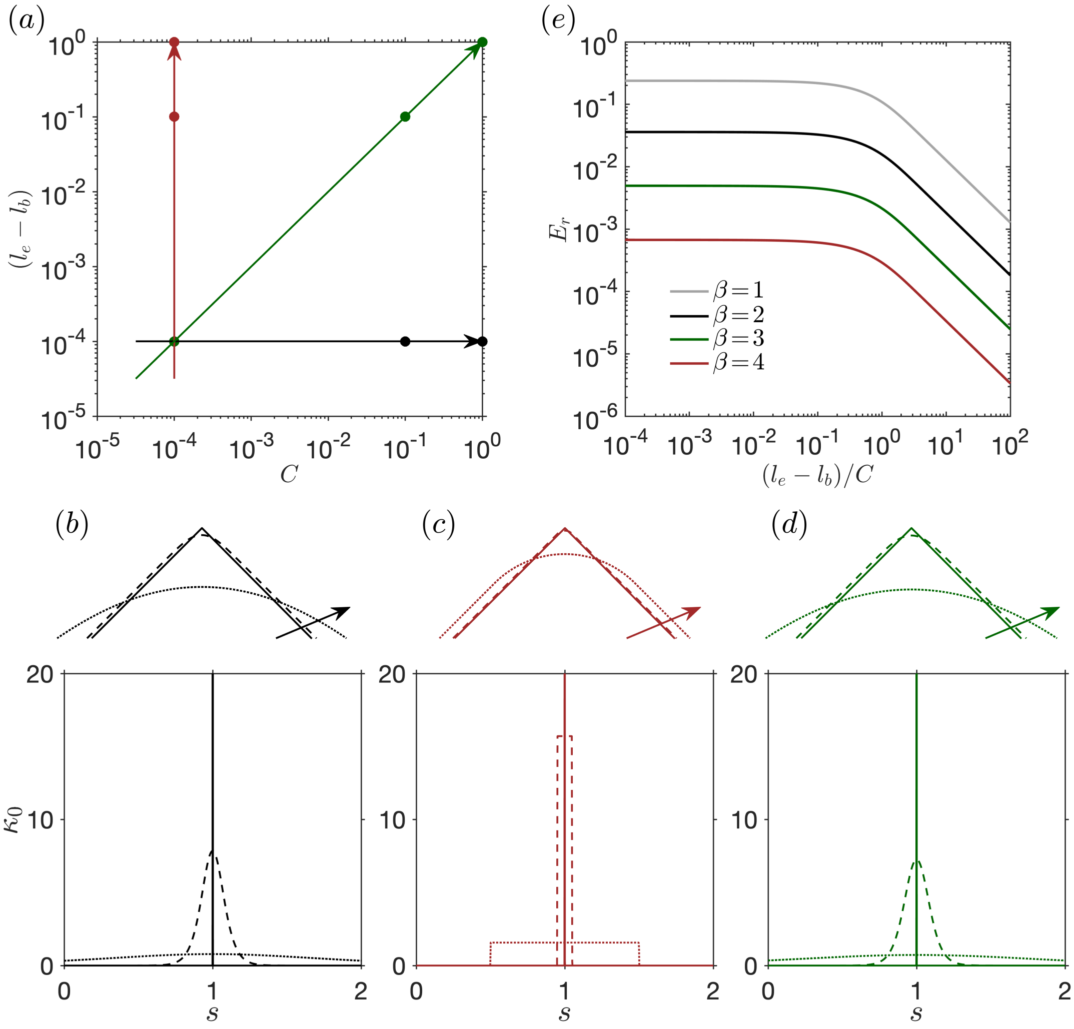

Fig. S1. Crease profiles and errors of crease angle with different geometric parameters C and $(l_e - l_b)$. (a) The three arrows represent different regions in C versus $(l_e - l_b)$ plane. (b-d) Crease profiles and their curvature distributions, with geometric parameters corresponding to the circles in (a) and the arrows following these in (a). (e) The error E_r (defined in Eq. (3)) with different β.

Without the loss of generality, we fix $\frac{l_b+l_e}{2}$ to 1, i.e. the crease is centered at $s=1$. The only remaining parameters affecting the crease profiles are (l_e-l_b) and C. Figure S1(b)-(d) display the crease profiles and rest curvatures with different parameter settings, corresponding to the colored arrows in Figure S1(a). We have assumed the length of the rod to be 2 (i.e. $s\in[0,2]$) and crease profiles are obtained by integrating the rest curvature κ_0.

Following the black arrow, we fix (l_e-l_b) to a small number 10^{-4} and vary C in the range $[10^{-4},1]$. With $C=10^{-4}$, the crease is extremely sharp which could be seen through the spike of the curvature. Increasing C makes the crease blunt and $C=1$ leads to a shallow arc that does not form the full crease angle. Following the brown arrow, C is fixed to a small number 10^{-4} with the increase of (l_e-l_b). In this case, the creases are uniformly bent when $(l_e-l_b)>>C$, e.g. the dashed and dotted renderings in Figure S1(c). Following the green arrow, we increase (l_e-l_b) and C simultaneously, resulting in creases similar to those in Figure S1(b). The two shallow dotted arcs in Figures S1(b) and S1(d) do not fully form the crease angle, because the length of the rod is less than the nominal crease length $(4C+l_e-l_b)$. For other cases, the resulting crease angle is very close to the target angle $\gamma=\pi/2$.

Next, we quantify the errors by calculating the deviation of the integral of $\Delta_C^{(l_b,l_e)}$ from unity in the region $[l_b-\beta C, l_e+\beta C]$,

$$E_r = 1-\int_{l_b-\beta C}^{l_e+\beta C}\Delta_C^{(l_b,l_e)}ds = 1-\frac{C}{l_e-l_b}\ln\frac{\cosh(\frac{l_e-l_b}{C}+\beta)}{\cosh\beta}, \qquad [3]$$

which shows that E_r only depends on the ratio $(l_e-l_b)/C$. Figure S1e reports E_r as a function of $(l_e-l_b)/C$ with different choices of β. When $(l_e-l_b)/C\to\infty$, $E_r\to 0$ no matter what the value of β. When $(l_e-l_b)=0$, E_r reaches a maximal value of $(1-\tanh\beta)$. For $\beta=2$, the maximal error is 3.6%. In other words, in the nominal crease region $[l_b-2C, l_e+2C]$, at least 96.4% of the crease angle has been formed. Increasing β reduces the error quickly. For $\beta=4$, E_r is less than 6.71×10^{-4}. We conclude that as long as the extremities of the rod are outside the nominal crease region, we will obtain an accurate crease angle.

2. NYC-skyline function

To characterize the NYC skyline in Figure S2 as a continuous function with C^∞, we use the boxcar feature of the Δ function (Eq. (1) in the main text). Figure S2 describes part of the New York City skyline through the following expression

$$NYC(x)=\sum_{i=1}^{53}\frac{f_i(x)}{2}\left[\tanh\left(\frac{x-l_{bi}}{C_i}\right)-\tanh\left(\frac{x-l_{ei}}{C_i}\right)\right], \qquad [4]$$

where $f_i(x)$ represents each segment of the piecewise continuous profile of the NYC skyline. We set C_i to be a small number 10^{-4} for all $i\in[1,53]$ and omit the prefactor $1/(l_{ei}-l_{bi})$, which leads to $\Delta=1$ in $x\in[l_{bi},l_{ei}]$ and $\Delta=0$ elsewhere. Here Δ works like a switch that only turns on $f_i(x)$ in $x\in[l_{bi},l_{ei}]$. Parameters and expressions of $NYC(x)$ are summarized in table S1.

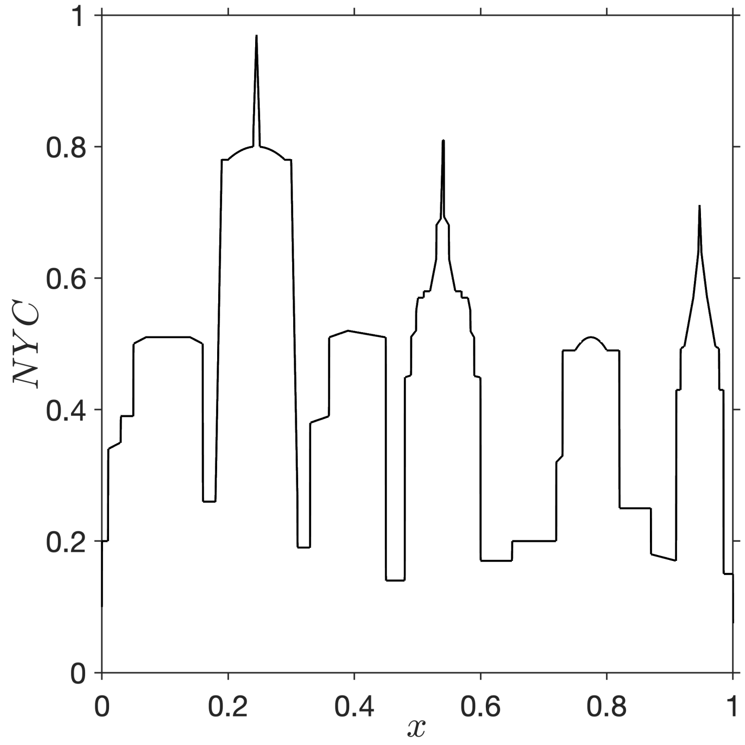

Fig. S2. NYC skyline, described by Eq. (4) as a continuous piece.

Table S1. Parameters and expressions used in the NYC skyline function in Eq. (4). C_i **is fixed to** 0.0001

i	l_{bi}	l_{ei}	$f_i(x)$	i	l_{bi}	l_{ei}	$f_i(x)$
1	0.0	0.01	0.2	28	0.542	0.544	$27.89 - 50s$
2	0.01	0.03	$0.335 + 0.5s$	29	0.544	0.55	$1.597 - 1.667s$
3	0.03	0.05	0.39	30	0.55	0.56	$3.38 - 5.s$
4	0.05	0.07	$0.475 + 0.5s$	31	0.56	0.57	0.58
5	0.07	0.14	0.51	32	0.57	0.58	0.57
6	0.14	0.16	$0.58 - 0.5s$	33	0.58	0.584	$3.47 - 5s$
7	0.16	0.18	0.26	34	0.584	0.59	$1.493 - 1.667s$
8	0.18	0.19	$-9.1 + 52s$	35	0.59	0.6	$0.569 - 0.2s$
9	0.19	0.30	0.78	36	0.6	0.65	0.17
10	0.20	0.29	$0.02 - 9.877(s - 0.245)^2$	37	0.65	0.72	0.2
11	0.24	0.245	$-7.18 + 30s$	38	0.72	0.73	$-0.4 + 1.s$
12	0.245	0.25	$7.52 - 30s$	39	0.73	0.82	0.49
13	0.3	0.31	$16.38 - 52s$	40	0.75	0.8	$0.02 - 32(s - 0.775)^2$
14	0.31	0.33	0.19	41	0.82	0.87	0.25
15	0.33	0.36	$0.27 + 0.333s$	42	0.87	0.91	$0.3975 - 0.25s$
16	0.36	0.39	$0.39 + 0.333s$	43	0.91	0.917	0.43
17	0.39	0.45	$0.585 - 0.167s$	44	0.917	0.923	$0.0345 + 0.5s$
18	0.45	0.48	0.14	45	0.923	0.937	$-4.383 + 5.286s$
19	0.48	0.49	$0.305 + 0.3s$	46	0.937	0.945	$-7.512 + 8.625s$
20	0.49	0.498	$-0.102 + 1.25s$	47	0.945	0.947	$-33.381 + 36s$
21	0.498	0.501	$-2.77 + 6.667s$	48	0.947	0.95	$23.439 - 24s$
22	0.501	0.51	0.57	49	0.95	0.959	$7.922 - 7.667s$
23	0.51	0.52	0.58	50	0.959	0.972	$6.029 - 5.692s$
24	0.52	0.53	$-2.02 + 5s$	51	0.972	0.978	$0.982 - 0.5s$
25	0.53	0.537	$-0.077 + 1.429s$	52	0.978	0.985	0.43
26	0.537	0.54	$-17.21 + 33.333s$	53	0.985	1.0	0.15
27	0.54	0.542	0.81				

3. Fabrication of creased annular strips

We 3D print regular pyramids to anneal creases, which fix the crease angle to γ, eliminate the residual stresses, and result in elastic creases (1–3). Figure S3a shows an example of the geometry of a regular pyramid with a triangular base and a superimposed creased annular strip with three creases. The geometry of the pyramid is fully determined by the ridge angle γ (i.e. the dihedral angle between two adjacent lateral faces), which prescribes the crease angle of creased annuli. Since γ is always larger than the internal angle of the polygonal base $(\pi - 2\pi/n_s)$, a pyramid with a polygon base of n_s sides can only be used for annealing creases with an angle larger than $(\pi - 2\pi/n_s)$.

With a prescribed ridge angle γ, the vertex angle of a lateral face ρ can be obtained as

$$\rho = 2\sin^{-1}\sqrt{\frac{\cos\gamma + \cos\frac{2\pi}{n_s}}{\cos\gamma - 1}}\,, \qquad [5]$$

In order to match the pyramid surface exactly, we laser cut annular strip with an effective length $\rho r_c n_s$ (excluding the gluing length of 2 mm), where r_c corresponds to the radius of the centerline. We use super glue to close the strip with the thickness of the gluing regions being slightly engraved in the cutting process. To achieve precise gluing of the two ends of annular strips, we 3D print a gadget consisting of a series of circular grooves, through which the two ends of the annular strip are pushed inside the groove to achieve good alignment (See Figure S3b). Even though the joint area almost doubles the thickness of the strip, its effects on the nonlinear mechanics of creased annuli are negligible due to its small length compared with the length of the annuli. After being annealed, the overcurvature of the stress-free creased annuli is $O_c = \rho n_s/(2\pi)$. We further cut the annealed strip and insert/remove annular arcs to adjust its overcurvature O_c. The inserted flat annular arcs are annealed together with the creased annular strips to make sure all the components of creased annuli have the same material properties.

Theoretically, the shape of creased annuli with a fixed cross section of the strip does not depend on the size of the experimental models, as long as the strip is slender (i.e. $L >> w$). However, models with a larger length will be more flexible and thus suffer more from gravity. On the other hand, smaller models have larger curvatures could experience plastic deformations. The sizes of the physical models (determined by radius r_c) are selected by trials and errors, such that the tabletop models do not suffer significantly from gravity, and at the same time, do not cause apparent plastic deformations to the strips. With the strip cross section fixed to w=5.08 mm and t=0.254 mm, we used r_c=40 mm for O_c=3, r_c=50 mm for O_c=1.5, and r_c=70 mm for O_c=0.7 in our tabletop models.

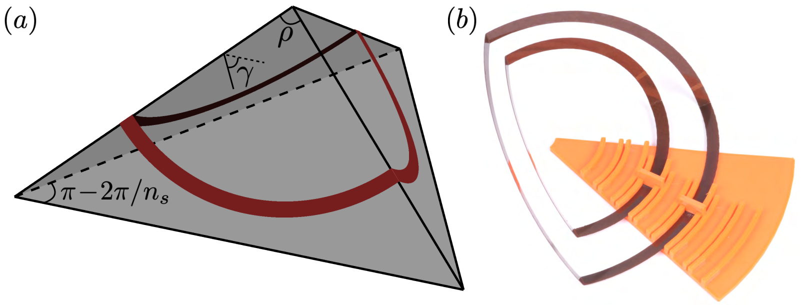

Fig. S3. 3D printed molds are used to construct precision tabletop models. (a) Geometry of a regular pyramid with a triangular base used to prescribe the crease angle of a creased annular strip to γ. (b) 3D printed circular grooves used for precise alignment in closing creased annuli with super glue.

4. Anisotropic rod model and nonlinear stability of equilibria

Anisotropic rod theory is normally used to model slender rods with the two dimensions of the cross section being in the same order of magnitude, e.g. $w \sim t$ for a strip with a rectangular cross section. Recent studies have shown that anisotropic rod model works quantitatively well for capturing the nonlinear mechanics of thin strips with w/t up to $O(10)$ (4–6). Here, we use anisotropic rod theory to solve the nonlinear mechanics of creased annuli. Throughout this study, we fix the aspect ratio of the cross section w/t to 20.

A local orthonormal right-handed material frame $(\boldsymbol{d}_1, \boldsymbol{d}_2, \boldsymbol{d}_3)$ is attached to the centerline of the strip, with $\boldsymbol{d}_3$ corresponding to the local tangent, and $\boldsymbol{d}_1$ and $\boldsymbol{d}_2$ aligned with the width w and thickness t, respectively (see Figure S4a). The kinematics of the material frame can be described as $\boldsymbol{d}_i' = \boldsymbol{\omega} \times \boldsymbol{d}_i$, with $\boldsymbol{\omega} = \kappa_1\boldsymbol{d}_1 + \kappa_2\boldsymbol{d}_2 + \tau\boldsymbol{d}_3$ and κ_1, κ_2 and τ corresponding to two bending curvatures and the twist, respectively. The internal forces and moments can be further resolved on the material frame as $\boldsymbol{N} = N_1\boldsymbol{d_1} + N_2\boldsymbol{d_2} + N_3\boldsymbol{d_3}$ and $\boldsymbol{M} = M_1\boldsymbol{d_1} + M_2\boldsymbol{d_2} + M_3\boldsymbol{d_3}$. Substituting the resolved forces and moments and the kinematics of the material frame into the equilibrium Equations (i.e. Eq. (3) in the main text), we have

$$\begin{gathered} N_1' - N_2\tau + N_3\kappa_2 = 0\,, N_2' + N_1\tau - N_3\kappa_1 = 0\,, N_3' + N_2\kappa_1 - N_1\kappa_2 = 0\,, \\ M_1' - M_2\tau - N_2 + M_3\kappa_2 = 0\,, M_2' + M_1\tau - M_3\kappa_1 + N_1 = 0\,, M_3' + M_2\kappa_1 - M_1\kappa_2 = 0\,. \end{gathered} \qquad [6]$$

We use linear constitutive laws $M_1 = EI_1(\kappa_1 - \kappa_{10})$, $M_2 = EI_2(\kappa_2 - \kappa_{20})$, and $M_3 = GJ\tau$, where E and G are the Young's modulus and shear modulus, respectively. EI_1, EI_2, and GJ are the two bending rigidities and the torsional rigidity, respectively. κ_{10} and κ_{20} correspond to the two bending curvatures of the rest configuration. Here, $\kappa_{10} = 2\pi O_c/L$ and $\kappa_{20} = \sum_{i=1}^{n_c}(\pi - \gamma_i)\Delta_{C_i}^{(l_{bi},l_{ei})}$, correspond to the geodesic curvature of the annular strip and the curvature of creases, respectively. Substituting the constitutive laws into Eq. (6), dividing the two sides of each equation by GJ, and defining the stiffness ratios $a = EI_1/(GJ)$ and $b = EI_2/(GJ)$, we have

$$
\begin{aligned}
&N_1' = (N_2\tau - N_3\kappa_2)l\,, N_2' = (-N_1\tau + N_3\kappa_1)l\,, N_3' = (-N_2\kappa_1 + N_1\kappa_2)l\,,\\
&a(\kappa_1' - \kappa_{10}') = (b(\kappa_2 - \kappa_{20})\tau - \tau\kappa_2 + N_2)l\,,\\
&b(\kappa_2' - \kappa_{20}') = (-a(\kappa_1 - \kappa_{10})\tau + \tau\kappa_1 - N_1)l\,,\\
&\tau' = (-b(\kappa_2 - \kappa_{20})\kappa_1 + a(\kappa_1 - \kappa_{10})\kappa_2)l\,,\\
&q_1' = \left(\tfrac{1}{2}(-q_2\tau + q_3\kappa_2 - q_4\kappa_1) + \mu q_1\right)l\,, q_2' = \left(\tfrac{1}{2}(q_1\tau + q_4\kappa_2 + q_3\kappa_1) + \mu q_2\right)l\,,\\
&q_3' = \left(\tfrac{1}{2}(q_4\tau - q_1\kappa_2 - q_2\kappa_1) + \mu q_3\right)l\,, q_4' = \left(\tfrac{1}{2}(-q_3\tau - q_2\kappa_2 + q_1\kappa_1) + \mu q_4\right)l\,,\\
&x' = 2(q_1^2 + q_2^2 - \tfrac{1}{2})l\,,\ y' = 2(q_2q_3 + q_1q_4)l\,,\ z' = 2(q_2q_4 - q_1q_3)l\,, s' = l\,,
\end{aligned}
\tag{7}
$$

where a prime denotes an $\bar{s}$ derivative ($\bar{s} = s/l \in [0,1]$) and (q_1, q_2, q_3, q_4) corresponds to the unit quaternions. In this study, the arc length s explicitly enters the equation through κ_{20} and the final ODE $s' = l$ turns the system into a standard boundary value problem. Following Healey and Mehta (7), the dummy parameter μ in Eq. (7) allows a consistent prescription of boundary conditions for quaternions. In numerical continuation, we treat μ as a free parameter and keep monitoring its value, which should be numerically zero (7). In our work, μ is found to be in the order of 10^{-14}.

For rods with a rectangular cross section composed of an elastically isotropic material, the bending and twisting stiffness are (8),

$$
EI_1 = \tfrac{1}{12}Ew^3t\,,\ EI_2 = \tfrac{1}{12}Ewt^3\,,\ GJ = \lambda Gwt^3 = \lambda\frac{E}{2(1+\nu)}wt^3\,,
\tag{8}
$$

which leads to

$$
a = \frac{EI_1}{GJ} = \frac{(1+\nu)}{6\lambda}\left(\frac{w}{t}\right)^2, b = \frac{EI_2}{GJ} = \frac{(1+\nu)}{6\lambda}\,.
\tag{9}
$$

Here ν is the Poisson's ratio, which is set to 0.33 in this study. λ depends on the aspect ratio of the cross section. For $w/t = 20$, we have $\lambda = 0.3228$ (8). Our formulation shows that material properties have minor influences on the nonlinear mechanics of creased annuli. First, the Young's Modulus does not appear in the normalized bending rigidities. Second, it is known that Poisson's ratio has minimal effects on the mechanics of anisotropic rods (6). We conclude that the nonlinear mechanics of creased annuli are mainly determined by the geometric parameters (i.e. the number of creases n_c, the crease angle γ, the overcurvature O_c, and the aspect ratio of the cross section of the strip w/t).

For the creased annuli, we clamp the middle of one segment at the origin of a Cartesian coordinate, with $\boldsymbol{d_1}$, $\boldsymbol{d_2}$, and $\boldsymbol{d_3}$ aligned to z, $-y$, and x direction, respectively (Figure S4a). The boundary conditions can be summarized as

$$
\begin{aligned}
&x(0) = 0\,, y(0) = 0\,, z(0) = 0\,, q_1(0) = 1\,, q_2(0) = 0\,, q_3(0) = 0\,, q_4(0) = 0\,,\\
&x(1) = 0\,, y(1) = 0\,, z(1) = 0\,, q_1(1) = -1\,, q_2(1) = 0\,, q_3(1) = 0\,, q_4(1) = 0\,, s(0) = 0\,.
\end{aligned}
\tag{10}
$$

Eq. (7) and Eq. (10) make a well-posed two point boundary value problem with fifteen unknowns N_1, N_2, N_3, κ_1, κ_2, τ, q_1, q_2, q_3, q_4, x, y, z, s, and μ. To solve the boundary value problem, we conduct numerical continuation through AUTO 07P, which uses orthogonal collocation and pseudo-arclength continuation to trace the solutions and detect bifurcations and limit points. We use a stress-free planar annulus in the $y - x$ plane as a starting point for continuation. The exact solution of the planar circle can be summarized as

$$
\begin{aligned}
&N_1 = 0\,, N_2 = 0\,, N_3 = 0\,, \kappa_1 = 2\pi/l\,, \kappa_2 = 0\,, \tau = 0\,, q_1 = \cos(\pi\bar{s})\,, q_2 = 0\,, q_3 = 0\,, q_4 = \sin(\pi\bar{s})\,,\\
&x = \tfrac{l}{2\pi}\sin(2\pi\bar{s})\,, y = \tfrac{l}{2\pi}[1 - \cos(2\pi\bar{s})]\,, z = 0\,, s = l\bar{s}\,; \quad \mu = 0\,, \gamma = \pi\,, O_c = 1\,.
\end{aligned}
\tag{11}
$$

To obtain the folded and inverted state, we first decrease κ_{10} by decreasing O_c ($\kappa_{10} = 2\pi O_c/l$), which leads to a pair of stable conical shapes bifurcated from the planar branch. Then we add crease angle to the bifurcated branches to obtain a folded and an inverted state, respectively. Finally, we systematically study how geometric parameters such as overcurvature and number of creases affect the nonlinear mechanics of creased annuli.

To determine the stability of an equilibrium solution $(\kappa_1, \kappa_2, \tau, N_1, N_2, N_3)$, we adopted a method from geometric mechanics to test the existence of conjugate point (9, 10), which applies to a single continuous rod with clamp-clamp boundary conditions. We solve the following matrix differential equations (9, 10),

$$
D' = FD\,, P' = QD + HP\,,
\tag{12}
$$

where a prime denotes an s derivative ($s \in [0, l]$ with l normalized to n_c). The coefficient matrices F, Q, and H can be written as (9, 10),

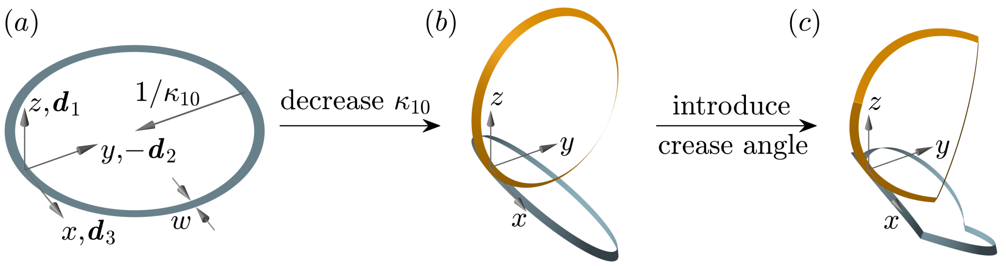

Fig. S4. Several continuation steps used to obtain the folded state and inverted state. (a) Start solution: a stress-free annular strip. (b) A pair of bifurcated conical shapes. (c) Folded (orange) and inverted states (blue).

$$F = \begin{bmatrix} 0 & M_2(\frac{1}{b}-\frac{1}{a})+\kappa_{20} & M_1(\frac{1}{b}-\frac{1}{a})-\kappa_{10} & 0 & 0 & 0 \\ M_2(1-\frac{1}{b})-\kappa_{20} & 0 & M_3(1-\frac{1}{b}) & 0 & 0 & 1 \\ M_1(\frac{1}{a}-1)+\kappa_{10} & M_3(\frac{1}{a}-1) & 0 & 0 & -1 & 0 \\ 0 & -\frac{N_2}{a} & \frac{N_1}{b} & 0 & \kappa_2 & -\kappa_1 \\ N_2 & 0 & -\frac{N_3}{b} & -\kappa_2 & 0 & \tau \\ -N_1 & \frac{N_3}{a} & 0 & \kappa_1 & -\tau & 0 \end{bmatrix} \quad [13]$$

$$Q = \begin{bmatrix} 1 & 0 & 0 & 0 & 0 & 0 \\ 0 & 1/a & 0 & 0 & 0 & 0 \\ 0 & 0 & 1/b & 0 & 0 & 0 \\ 0 & 0 & 0 & 0 & 0 & 0 \\ 0 & 0 & 0 & 0 & 0 & 0 \\ 0 & 0 & 0 & 0 & 0 & 0 \end{bmatrix}, H = \begin{bmatrix} 0 & \kappa_2 & -\kappa_1 & 0 & 0 & 0 \\ -\kappa_2 & 0 & \tau & 0 & 0 & 0 \\ \kappa_1 & -\tau & 0 & 0 & 0 & 0 \\ 0 & 0 & 0 & 0 & \kappa_2 & -\kappa_1 \\ 0 & 0 & 1 & -\kappa_2 & 0 & \tau \\ 0 & -1 & 0 & \kappa_1 & -\tau & 0 \end{bmatrix}, \quad [14]$$

where $\kappa_1 = \frac{M_1}{a} + \kappa_{10}$, $\kappa_2 = \frac{M_2}{b} + \kappa_{20}$, and $\tau = M_3$ (notice that torsional stiffness has been normalized to unity). Together with the initial conditions $D(0) = I_{6\times6}$ and $P(0) = 0_{6\times6}$, we obtain an initial value problem that is solved with MATLAB. If the solution of Eq. (12) satisfies $\det(P) \neq 0$ for all $s \in (0, l]$, then the equilibrium is stable. If $\det(P) = 0$ for some $s \in (0, l]$, then the equilibrium is unstable. If $\det(P) \neq 0$ for all $s \in (0, l)$ and $\det(P(l)) = 0$, then we cannot conclude the stability and higher order variations of the energy functional need to be considered (9, 10).

Figure S5(a-b) and S5(c-d) summarized the solutions and conjugate point tests of creased annuli with six creases ($l = 6$) and two creases ($l = 2$), respectively. The bifurcation curves are presented in the energy ε versus overcurvature O_c plane, with $(C_i, l_{ei} - l_{bi})$ fixed to $(0.002, 4\times10^{-7})$. Some of the test results $\det(P)$ are multiplied with a factor, which does not affect the stability information. For example, in Figure S5(b), the black curve represents $10^{-3}\times$ the test result of the solution ⧫ in S5(a). In all the tests, unstable solutions contain at least one conjugate point (where the scaled $\det(P)$ crosses zero) and stable solutions are free of conjugate points in $s \in (0, l]$. Right at a critical point such as a fold or bifurcation point, $\det P(l) = 0$ and a conjugate point is located exactly at $s = l$ end. For example, the ● and ● in Figure S5(a-b) and the ● in Figure S5(c-d).

5. Different regularized Dirac delta functions

Here, we use the following RDDFs to describe the local geometry of a crease centered at $s = 1$

$$\delta_1 = \frac{1}{\pi}\frac{C_1}{(s-1)^2 + C_1^2}, \delta_2 = \frac{1}{2C_2}\frac{1}{[1 + (\frac{s-1}{C_2})^6]^{7/6}}, \delta_3 = \frac{1}{2}(\delta_1 + \delta_2). \quad [15]$$

The rest curvature of a crease with target angle γ can be described as $(\pi - \gamma)\delta_i$. Here C_i controls the sharpness of the crease and a perfect crease with C^0 continuity is achieved by taking the limit $C_i \to 0$. C_i is obtained by setting the nominal crease length of δ_i to be the same with Δ. Specifically, in Figure S6a, we have $\int_{0.996}^{1.004} \delta_i ds = 96.4\%$ for all $i = 1, 2, 3$, which is also true for Δ with $C = 0.002$ and $(l_e - l_b) = 2\times10^{-4}C$.

Figure S6(a) shows the curvature distribution of different RDDFs with γ fixed to 0.5π. While the curvature clearly contains a similar localized feature around the crease at $s = 1$ (shown as spikes with different heights), the inset shows the significant differences of the curvature in the nominal crease region. The crease profiles generated by these δ functions can be obtained by integrating the curvature, which are shown together in Figure S6(b) for the global shape and Figure S6(c) for the local crease geometry. We remark that even though the local crease geometry varies significantly across different RDDFs, no notable differences are found in the global shapes. Intuitively, we would expect that if the various right-angle rods in Figure S6(b) are subject to the same loading, the nonlinear mechanics responses would be the same, even though the rods may contain very different local deformations around the crease region. Figure S6(d-e) reports the curvature distribution and the corresponding crease profiles by scaling the size of the creases in Figure S6(a) up to 150 times. In this case, significant differences among

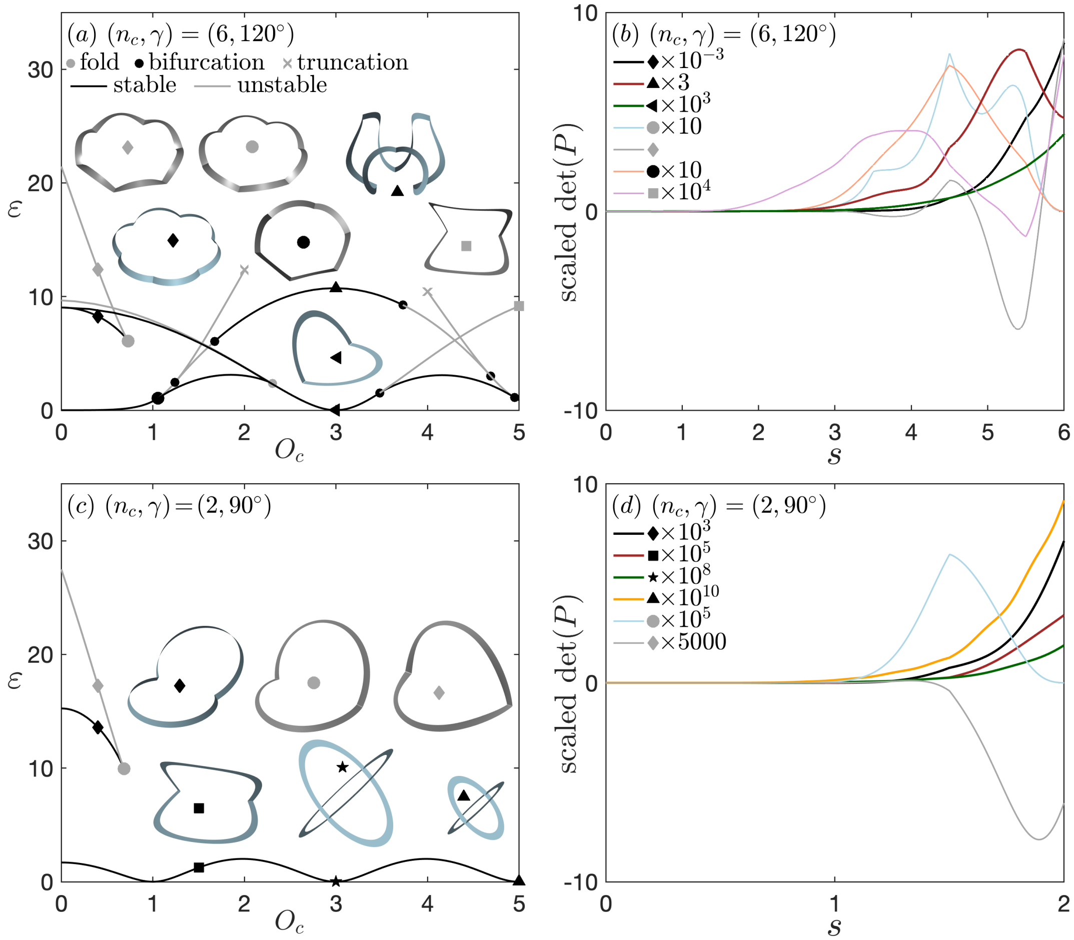

Fig. S5. Bifurcation curves (black: stable; grey: unstable) and examples of conjugate point tests of creased annuli. (a) Solutions with six creases. (b) Conjugate point tests of several solutions in (a). (c) Solutions with two creases. (d) Conjugate point tests of several solutions in (c).

different creases are observed in the global shapes, and we would expect different mechanical behaviors of these structures subjected to identical loading conditions.

In the following, we use an example to demonstrate that as long as creases are sharp, any RDDF can be implemented to solve the nonlinear mechanics of creased structures. Figure S7 displays numerical results of creased annuli with (n_c, γ) fixed to $(2, 90°)$ and the crease described by different RDDFs in Eq. (15). Figure S7(a-b) include numerical results of sharp creases whose geometric parameters are the same as those in Figure S6(a). With sharp creases, the difference in the elastic energy of different crease profiles is negligible and the deformed shapes almost coincide with each other, demonstrated through the four square markers and their corresponding renderings. This is also true for the strains τ and $(\kappa_2 - \kappa_{20})$ of the solutions marked by squares, reported in Figure S7(b). The insets in Figure S7(b) shows that notable differences only exist around the crease region. In contrast, Figures S7(c-d) present numerical results of blunt creases sharing the same geometric parameters with those in Figure S6(d). With different crease profiles, significant differences are found in the elastic energy ε, deformed shapes (the renderings in Figure S7(c)), and strains (Figure S7(d); corresponding to the squares in Figure S7(c)). We conclude that any RDDF could be used to model sharp creases without causing notable differences in the nonlinear mechanics (large deformations, stability, etc.) of creased strips. The Δ function proposed in the current study could address different types of discontinuities because it contains both Heaviside and Dirac-delta features.

6. Slender rods with material discontinuities and nonlinear material properties

This study mainly focuses on the description of the sharp geometry of creases and assume the creased regions contain the same elastic materials as the rest part. However, creases could be more complicated in terms of both geometry and material properties. For example, without annealing, creases could follow a complex relaxation mechanism and have a highly nonlinear behavior (11). In order to promote the formation of crease, the thickness and width of the creased regions can be reduced (12–14), which leads to nonuniform cross section.

Here, we show an example using our framework to model a ribbon with the creased regions having a reduced width and the material following an approximate elastic-plastic behavior. We choose the example studied in (12), where the authors show that both local reduction of width and plastic deformations can promote the formation of creases. Figure S8a shows the

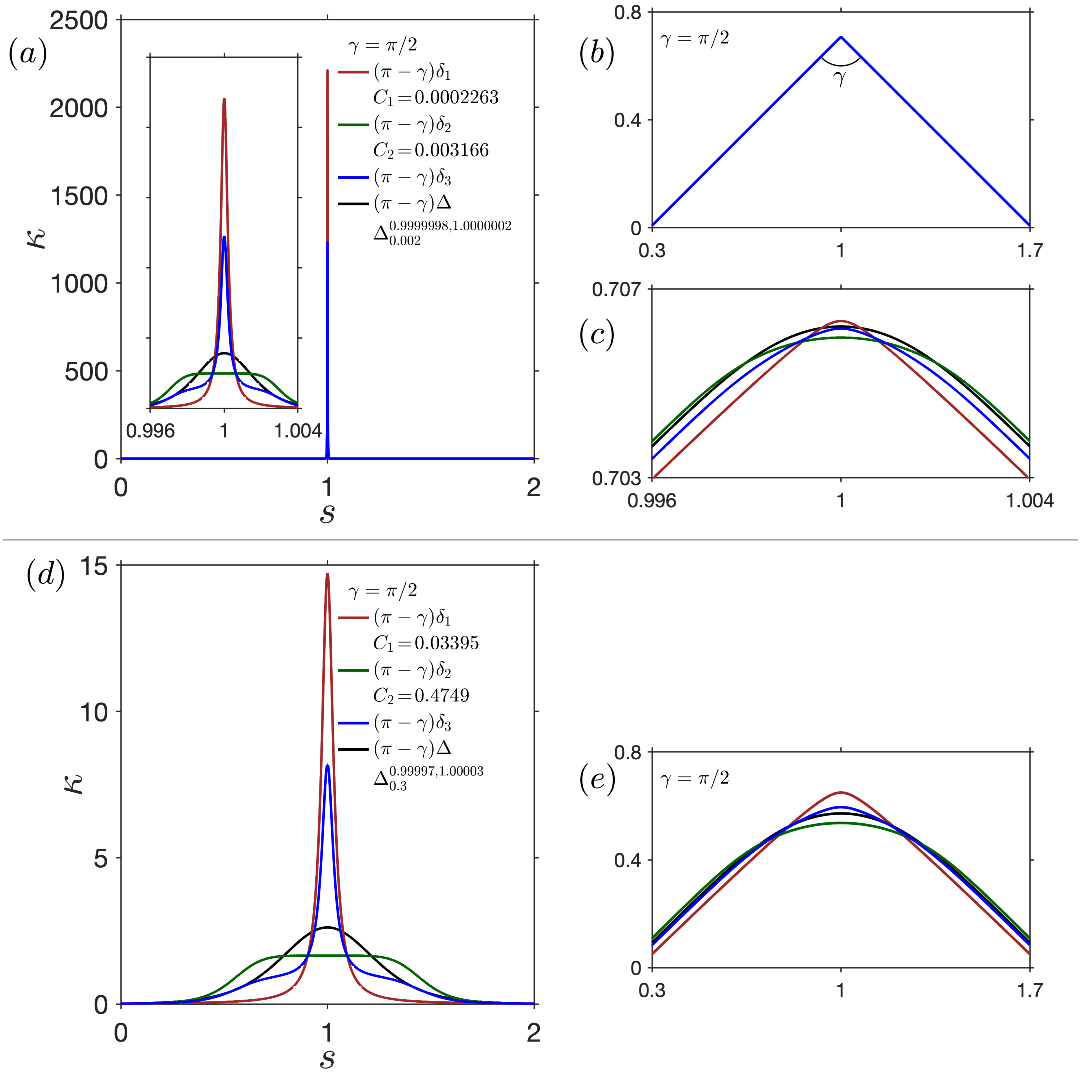

Fig. S6. Different RDDFs (Eq. (15)) are used to describe the local geometry of a crease centered at $s = 1$ with a normalized length 2. (a) Curvature distribution of a sharp crease with $\gamma = \pi/2$. (b) The corresponding rod has no notable differences. (c) A blowup of the local crease region shows significant differences across different RDDFs. (d) Creases that are 150 times the size of (a) are obtained by scaling the geometric parameters accordingly. (e) The resulted rod geometries from (d) contains significant differences.

geometry of the ribbon with both ends clamped and one end subject to a compressive displacement δ (12). The total length of the rod is normalized to unity. w_c and w represent the reduced width at a crease and the width elsewhere, respectively. l_c corresponds to the length of the creases, located in the regions $[0, l_c]$, $[l_c + 0.5(1-3lc), 2l_c + 0.5(1-3lc)]$, and $[1-l_c, 1]$. Upon compression, the system buckles out of plane, as shown in Figure S8b. An orthonormal material frame $(\boldsymbol{d}_2, \boldsymbol{d}_3)$ is attached to the centerline of the rod; θ measures the angle between the unit tangent $\boldsymbol{d}_3$ and the horizontal direction.

For uniform rods made of linear elastic materials, the internal moment M follows Hooke's law, i.e. $M(\kappa) = EI\kappa$. Here E and I corresponds to the Young's Modulus and the second moment of area, respectively. For general case, we assume

$$M(\kappa, s) = B(s)f(\kappa)\,. \tag{16}$$

Through our continuous description of an arbitrary piecewise continuous function (see SI Appendix, section 2), $B(s)$ can account for the abrupt variation of cross sections, material thickness and material properties along the arc length and $f(\kappa)$ can characterize an arbitrary nonlinear dependence of bending moment on the curvature. In Figure S8a, the abrupt change of the width leads to nonsmooth $B(s)$, which can be described as the following continuous function

$$\begin{aligned} B(s) &= \frac{w_c}{w} + (1.0 - w_c/w)(l_{e1} - l_{b1})\Delta_C^{(l_{b1}, l_{e1})} + (1.0 - w_c/w)(l_{e2} - l_{b2})\Delta_C^{(l_{b2}, l_{e2})} \\ \text{with } l_{b1} &= l_c, l_{e1} = l_c + \frac{(1-3l_c)}{2}, l_{b2} = 2l_c + \frac{(1-3l_c)}{2}, l_{e2} = 1 - l_c\,. \end{aligned} \tag{17}$$

We normalize $B(s)$ at a full-width cross section to unity. Figure S8c shows an example of Eq. (17). When $C \to 0$, $B(s)$ will

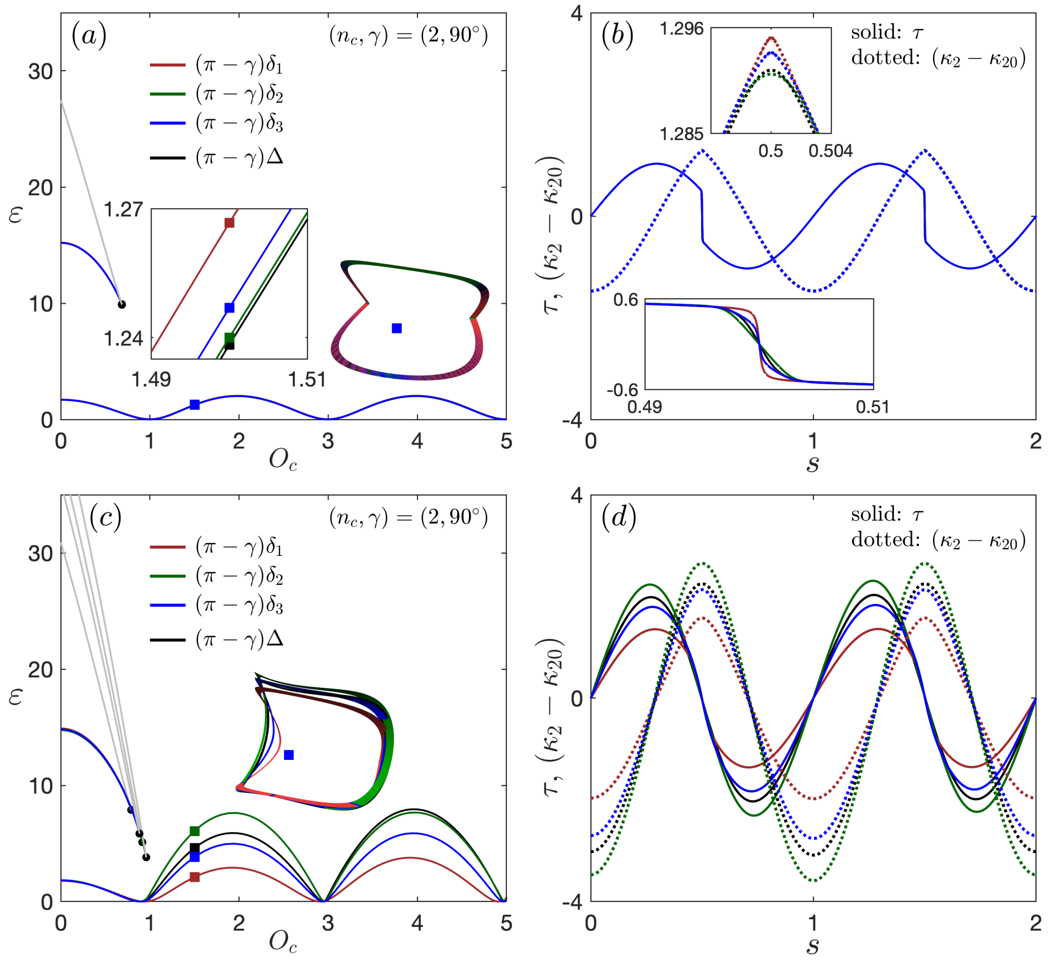

Fig. S7. Numerical results of creased annular strips with (n_c, γ) fixed to $(2, 90^\circ)$ and the creases described by different RDDFs in Eq. (15). (a) Solution curves of sharp creases with the same geometric parameters as in Figure S7(a). (b) Strains of several equilibria corresponding to the squares in (a). (c) Solution curves of blunt creases with the same geometric parameters as in Figure S7(d). (b) Strains of several equilibria corresponding to the squares in (c).

approach the ideal case. Here, we set $C = l_c/100$, which corresponds to a sharp jump of the cross section. Figure S8d reports the profile of a hyperbolic tangent function plus a small linear term, mimicking an elastic plastic constitutive law.

We adopt the *elastica* theory to solve this problem. The governing equations correspond to the planar version of Eq. (7) and can be written as

$$
\begin{aligned}
&N_2' = N_3\kappa\,, N_3' = -N_2\kappa\,,\\
&\kappa' = (N_2 - B'f)/(Bf_\kappa)\,,\\
&\theta' = \kappa\,, x' = \cos\theta\,, y' = \sin\theta\,, s' = 1\,,
\end{aligned}
\qquad [18]
$$

where a prime represents a partial derivative with respect to arc length s and $f_\kappa = df/d\kappa$. N_2 and N_3 corresponds to the internal forces resolved on the material frame $(\boldsymbol{d}_2, \boldsymbol{d}_3)$. The differential equation for κ is obtained by substituting $M = Bf$ into the moment balance equation $M' = N_2$. In addition, we introduce one more trivial equation $s'=1$ because the arc length explicitly enters the equation through M'. The boundary conditions can be summarized as

$$
x(0) = 0, y(0) = 0, \theta(0) = 0, x(1) = 1 - \delta, y(1) = 0, \theta(1) = 0, s(0) = 0\,. \qquad [19]
$$

We conduct numerical continuation to solve the two point boundary value problem Eq. (17)-Eq. (18). The second row in Figure S8 presents the numerical results with $f(\kappa) = \kappa$. Figure S8e reports the force displacement curves with different w_c/w and l_c fixed to 0.01. The solid circles correspond to the critical load P_b where the structure buckles out of plane. With $w_c/w = 1$, we obtain a buckling load 39.4784, which matches exactly with the Euler buckling load $4\pi^2$. Decreasing w_c/w reduces the buckling load.

Figure S8f shows a series of renderings from the solutions in Figure S8e. Decreasing the crease width w_c/w leads to more pronounced localized deformation at the creases and thus makes the facets flatter. However, even with a very small crease width $w_c/w = 0.01$, bending of the facets are still noticeable. Figure S8g shows the loci of the buckling point P_b (normalized

by Euler buckling load) in the w_c/w versus $P_b/(4\pi^2)$ plane for different crease length l_c. These loci curves are obtained by conducting two parameter continuation. Generally speaking, to achieve the same buckling load, a longer crease needs a larger width.

The third row reports numerical results with $f(\kappa)$ following a nonlinear elastic behavior, which mimics a material that approaches a plastic plateau stress after significant bending (see Figure S8d). We wish to emphasize that here we are not attempting to model any unloading or recovery of the crease and only consider the forward formation of the crease, so that the distinction between elastic and plastic response is not an issue. Figure S8h reports the force displacement curves with different w_c/w. Generic softening behaviors are observed, i.e. the load decreases after the buckling point. Once the plateau moment is reached at the crease, bending deformation quickly localizes there. Figure S8i shows a series of renderings from the solutions in Figure S8h. Compared with Figure S8f, the results in Figure S8i clearly show that the introduction of a plateau bending moment can promote the formation of the crease, which behaves like a plastic hinge that can sustain large rotations with an almost constant bending moment. Notice that without much reduction of the crease width (see the case with $w_c/w = 0.9$ and 0.5 in Figure S8i), only introducing an elastic-plastic-like material law cannot guarantee flat facets.

Figure S8j reports the curvature distribution of a configuration with $w_c/w = 0.1$ and $\delta = 0.5$. It is clearly that curvatures are highly localized at the creases. In addition, the two insets show vertical jump of curvature at the interface connecting the crease and the rest part of the ribbon (e.g. at $s = 0.01$, $s = 0.495$, and $s = 0.505$), which is due to the fact that bending moment is continuous, but the bending stiffness jumps at the interface.

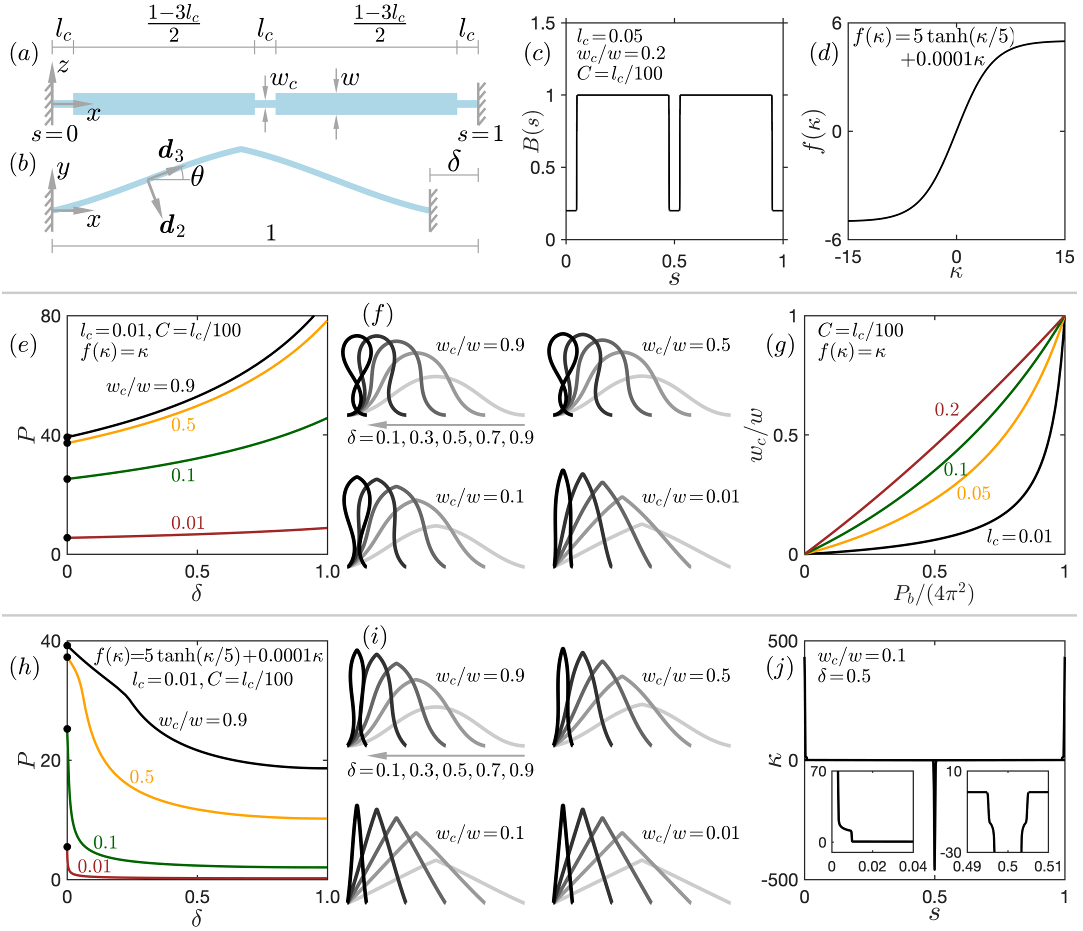

Fig. S8. The formation of a crease. (a) The geometry of the ribbon in the rest configuration. The width is locally reduced at the mid and the two ends to promote folding. (b) The ribbon buckles out of plane with localized deformation at the width-reduction regions. (c) $B(s)$ accounts for the variation of width in (a) through a continuous function (Eq. (17)). (d) $f(\kappa)$ follows a hyperbolic tangent plus a small linear term, mimicking an elastic-plastic constitutive law. The second row reports numerical results with $f(\kappa) = \kappa$. (e) Force-displacement curves with different w_c/w. The solid circles correspond to the critical load P_b where the structure buckles out of plane. (f) Renderings of solutions in (e). (g) Loci of the critical load P_b in the w_c/w versus $P_b/(4\pi^2)$ plane for different crease length l_c. The third row reports the numerical results with $f(\kappa)$ mimicking an elastic-plastic behavior. (h) Force-displacement curves with different w_c/w. (i) Renderings of solutions in (h). (j) Curvature distribution of a configuration with $w_c/w = 0.1$ and $\delta = 0.5$.

7. Continuous description of 2D surfaces with geometric discontinuities

The creased annular strips studied in this work contain 1D discontinuities along the arc length of the strip. Here, we show that a hyperbolic tangent could be used to describe 2D surfaces with discontinuities. 2D steps can be described through the following equation

$$H(r(x,y)) = \frac{1}{2}\left[\tanh\left(\frac{r(x,y)}{C}\right) + 1\right], \quad [20]$$

where r is a real valued function of x and y and C controls the sharpness of the step. When $C \to 0$, $H(x,y) = 1$ for $r(x,y) > 0$ and $H(x,y) = 0$ elsewhere. The domain $r(x,y) > 0$ will be referred to as the base of the 2D step and the curve $r(x,y) = 0$ represents its boundary. Figures S9a and S9b displays a half-plane based step S with $r = 3x + 2y - 1$ and a dumbbell based step DB with $r = x^4 - x^6 - y^2 + 0.01$, respectively. The product of several half-plane based step functions $H(r_i)$ can be used to describe a step with a convex polygonal base

$$P(x,y) = \prod_{i=1}^{n_s} H(r_i(x,y)), \quad [21]$$

where n_s represents the number of sides of the polygonal base. The i-th side of the polygon base is part of the line $r_i(x,y) = 0$. For a point inside the polygon (x_p, y_p), we always have $r_i(x_p, y_p) > 0$. Figure S9c displays a step with a convex polygonal base, described by the following function

$$\begin{aligned} P_1 = &[0.5(\tanh((0.5x - 0.1)/C) + 1)][0.5(\tanh((-0.1x - 0.4y + 0.3)/C) + 1)] \\ &[0.5(\tanh((-0.3x - 0.1y + 0.24)/C) + 1)][0.5(\tanh((-0.1x + 0.5y - 0.08)/C) + 1)]. \end{aligned} \quad [22]$$

Steps with a concave polygonal base can be obtained by adding those associated to the convex partitions of the concave base. Figure S9d presents an example combining the geometry in Figure S9(c) and an additional step with a convex polygonal base, described by the following function,

$$\begin{aligned} P_2 = P_1 + &[0.5(\tanh((-0.6y + 0.42)/C) + 1)][0.5(\tanh((-0.1x + 0.2y - 0.06)/C) + 1)] \\ &[0.5(\tanh((0.1x + 0.4y - 0.3)/C) + 1)]. \end{aligned} \quad [23]$$

Multiplying $H(x,y)$ by an arbitrary function allows us to generate a new function that equals to the original function within the base of H and vanishes elsewhere. Figure S9e displays a single dimple on a thin sheet, described by

$$D_1 = \left[0.5(\tanh((0.25 - x^2 - y^2)/C) + 1)\right]\left[1.6(0.25 - x^2 - y^2)\right]. \quad [24]$$

Combining several such functions, we are able to decorate multiple dimples of different geometries in a thin sheet. Figure S9f presents an example described by the following function

$$\begin{aligned} D_2 = &\left[0.5(\tanh((0.17^2 - x^2 - y^2)/C) + 1)\right]\left[6(0.17^2 - x^2 - y^2)\right] \\ &+ \left[0.5(\tanh((0.15^2 - (x-0.5)^2 - (y-0.5)^2)/C) + 1)\right]\left[7(0.15^2 - (x-0.5)^2 - (y-0.5)^2)\right] \\ &+ \left[0.5(\tanh((0.15^2 - (x+0.5)^2 - (y+0.5)^2)/C) + 1)\right]\left[7(0.15^2 - (x+0.5)^2 - (y+0.5)^2)\right] \\ &+ \left[0.5(\tanh((0.15^2 - (x+0.5)^2 - (y-0.5)^2)/C) + 1)\right]\left[7(0.15^2 - (x+0.5)^2 - (y-0.5)^2)\right] \\ &+ \left[0.5(\tanh((0.15^2 - (x-0.5)^2 - (y+0.5)^2)/C) + 1)\right]\left[7(0.15^2 - (x-0.5)^2 - (y+0.5)^2)\right] \\ &+ \left[0.5(\tanh((0.25^2 - (x-0.5)^2 - y^2)/C) + 1)\right]\left[3(0.25^2 - (x-0.5)^2 - y^2)\right] \\ &+ \left[0.5(\tanh((0.25^2 - (x+0.5)^2 - y^2)/C) + 1)\right]\left[3(0.25^2 - (x+0.5)^2 - y^2)\right] \\ &+ \left[0.5(\tanh((0.25^2 - x^2 - (y-0.5)^2)/C) + 1)\right]\left[3(0.25^2 - x^2 - (y-0.5)^2)\right] \\ &+ \left[0.5(\tanh((0.25^2 - x^2 - (y+0.5)^2)/C) + 1)\right]\left[3(0.25^2 - x^2 - (y+0.5)^2)\right]. \end{aligned} \quad [25]$$

The above continuous descriptions of 2D surfaces with discontinuities could be useful in numerical modeling of shape-morphing metasheets (15, 16). In addition, H can be used to approximate a complicated surface by partitioning it, describing each subdomain with a continuous function, and combining these functions together. Figure S10 displays the external surface of the Empire State Building, characterized by the following function

$$\begin{aligned} ESB(x,y) = &\sum_{i=1}^{24} \frac{z_i}{2^4}\left[\tanh\left(\frac{x - x_{i1}}{C}\right) + 1\right]\left[\tanh\left(\frac{x_{i2} - x}{C}\right) + 1\right]\left[\tanh\left(\frac{y - y_{i1}}{C}\right) + 1\right]\left[\tanh\left(\frac{y_{i2} - y}{C}\right) + 1\right] \\ &+ \frac{0.3}{2}\left[\tanh\left(\frac{0.01^2 - (x-0.5)^2 - (y-0.5)^2}{C}\right) + 1\right] \end{aligned} \quad [26]$$

where the first 24 functions contain steps with rectangular bases $[x_{i1}, x_{i2}] \times [y_{i1}, y_{i2}]$ and heights z_i. Their values are summarized in table S2. The cylindrical antenna is modeled by the last term, corresponding to a circle based step. The plot in Figure S10a is obtained with $C = 0.00001$, which plays an important role in determining the sharpness of the geometric discontinuity. Figures S10(b-d) report this effect by rendering Eq. (26) with larger values of C.

Table S2. Parameters of the rectangle based step functions used to describe the Empire State Building in Figure S10 (see Eq. (26)).

	x_{i1}	x_{i2}	y_{i1}	y_{i2}	z_i	i	x_{i1}	x_{i2}	y_{i1}	y_{i2}	z_i
1	0.1	0.9	0.3	0.7	0.1	13	0.35	0.45	0.4	0.6	0.9
2	0.2	0.3	0.4	0.6	0.3	14	0.55	0.65	0.4	0.6	0.9
3	0.7	0.8	0.4	0.6	0.3	15	0.45	0.55	0.43	0.57	1.2
4	0.3	0.35	0.35	0.65	0.4	16	0.365	0.45	0.415	0.585	0.2
5	0.65	0.7	0.35	0.65	0.4	17	0.55	0.635	0.415	0.585	0.2
6	0.35	0.4	0.35	0.4	0.4	18	0.38	0.45	0.43	0.57	0.1
7	0.35	0.4	0.6	0.65	0.4	19	0.55	0.62	0.43	0.57	0.1
8	0.6	0.65	0.35	0.4	0.4	20	0.4	0.6	0.45	0.55	0.05
9	0.6	0.65	0.6	0.65	0.4	21	0.42	0.58	0.46	0.54	0.02
10	0.3	0.35	0.45	0.55	0.1	22	0.43	0.57	0.47	0.53	0.01
11	0.65	0.7	0.45	0.55	0.1	23	0.44	0.56	0.48	0.52	0.01
12	0.35	0.65	0.4	0.6	0.5	24	0.45	0.55	0.49	0.51	0.01

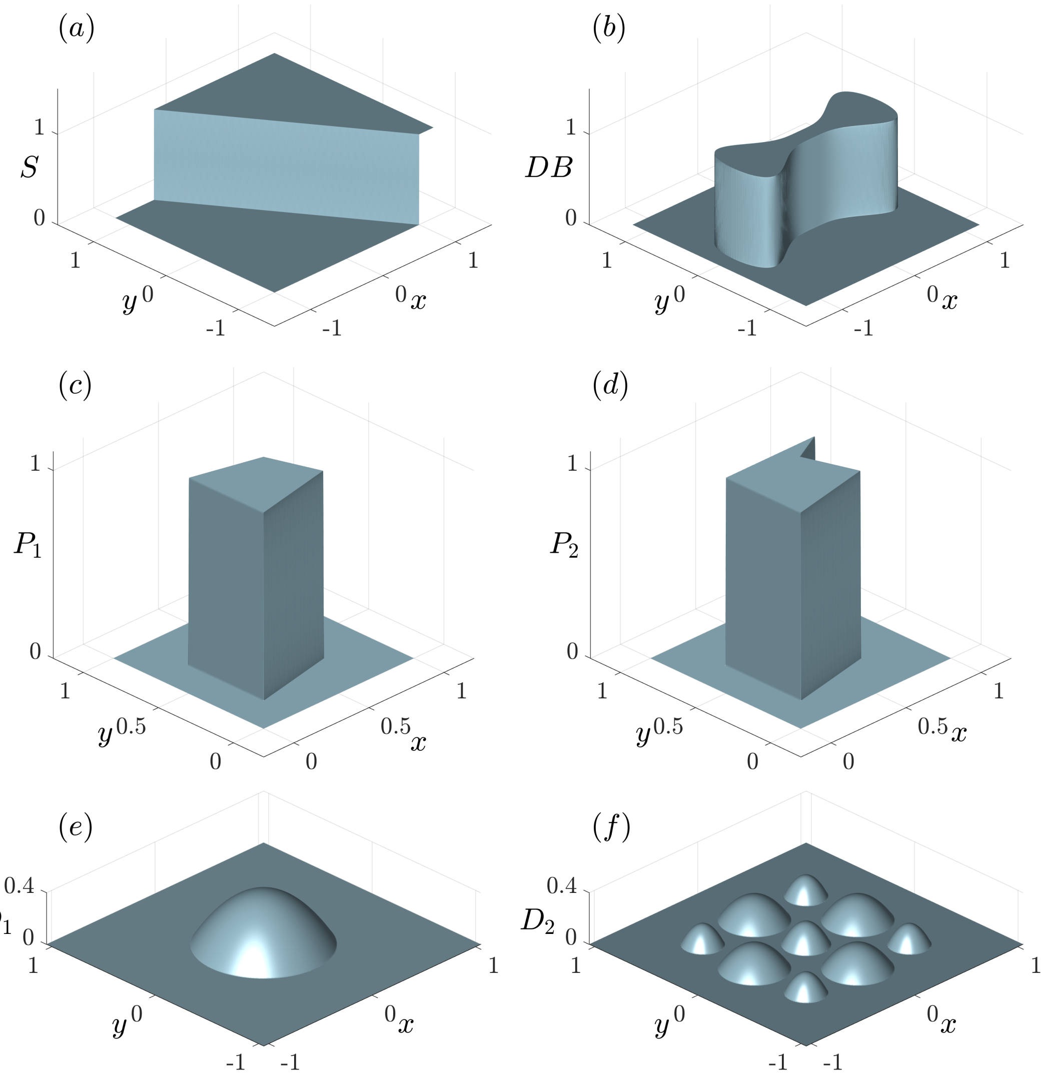

Fig. S9. Different geometries based on the function in Eq. (20) with C fixed to 0.0002. (a) A half-plane based step S. (b) A dumbbell based step DB. (c) A convex-polygon based step P_1. (d) A concave-polygon based step P_2. (e) A single dimple described by Eq. (24). (f) Dimples of different geometries in a thin sheet, described by Eq. (25).

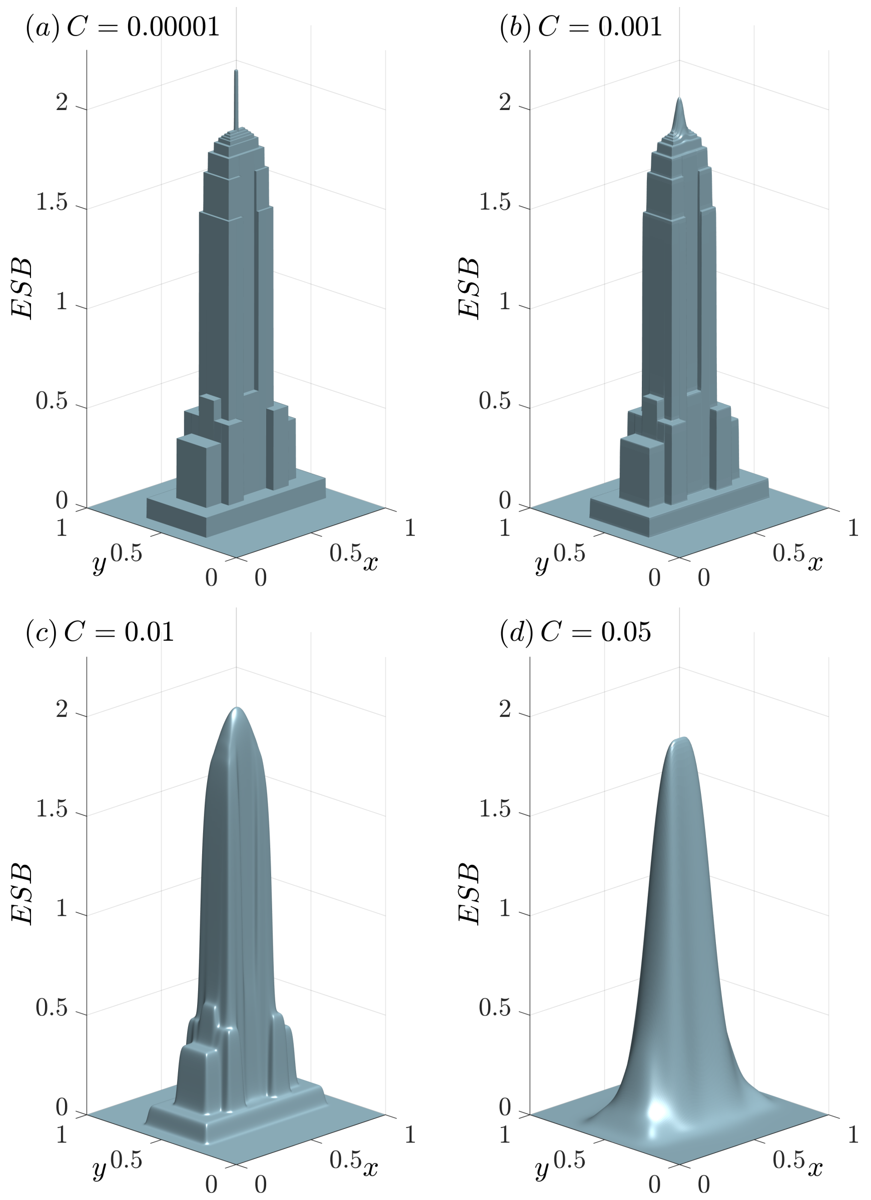

Fig. S10. The external geometry of the Empire State Building characterized as a continuous surface by Eq. (26) with (a) $C=0.00001$, (b) $C=0.001$, (c) $C=0.01$, and (d) $C=0.05$.

SI Movies

Movie S1. Generic bistability of creased annuli with small overcurvature. This movie demonstrates generic bistability in creased annuli with $O_c = 0.7$. The four geometric parameters (n_c, O_c, γ, r_c) correspond to the number of creases, overcurvature of the flat annuli, crease angle, and the radius of curvature of the flat annuli, respectively.

Movie S2. Looping behaviors of creased annuli with large overcurvature. This movie demonstrates looping behaviors of creased annuli with large overcurvature. The four geometric parameters (n_c, O_c, γ, r_c) correspond to the number of creases, overcurvature of the flat annuli, crease angle, and the radius of curvature of the flat annuli, respectively. The model with five creases can be folded into a star configuration and the model with three creases can be folded into three loops, with each loop containing one crease.

References

1. T Jules, F Lechenault, M Adda-Bedia, Local mechanical description of an elastic fold. *Soft Matter* **15**, 1619–1626 (2019).
2. Y Klett, Paleo: Plastically annealed lamina emergent origami in *ASME 2018 International Design Engineering Technical Conferences and Computers and Information in Engineering Conference*. Vol. 51814, p. V05BT07A062 (2018).
3. B Sargent, et al., Heat set creases in polyethylene terephthalate (pet) sheets to enable origami-based applications. *Smart Mater. Struct.* **28**, 115047 (2019).
4. D Riccobelli, G Noselli, A DeSimone, Rods coiling about a rigid constraint: helices and perversions. *Proc. R. Soc. A* **477**, 20200817 (2021).
5. DE Moulton, P Grandgeorge, S Neukirch, Stable elastic knots with no self-contact. *J. Mech. Phys. Solids* **116**, 33–53 (2018).
6. T Yu, JA Hanna, Bifurcations of buckled, clamped anisotropic rods and thin bands under lateral end translations. *J. Mech. Phys. Solids* **122**, 657–685 (2019).
7. TJ Healey, PG Mehta, Straightforward computation of spatial equilibria of geometrically exact cosserat rods. *Int. J. Bifurc. Chaos* **15**, 949–965 (2005).
8. S Timoshenko, JN Goodier, *Theory of Elasticity.* (McGraw-Hill), (1951).
9. T Bretl, Z McCarthy, Quasi-static manipulation of a kirchhoff elastic rod based on a geometric analysis of equilibrium configurations. *The Int. J. Robotics Res.* **33**, 48–68 (2014).
10. A Borum, T Bretl, When is a helix stable? *Phys. Rev. Lett.* **125**, 088001 (2020).
11. B Thiria, M Adda-Bedia, Relaxation mechanisms in the unfolding of thin sheets. *Phys. Rev. Lett.* **107**, 025506 (2011).
12. Y Shi, Y Zhang, Plasticity-induced origami for assembly of three dimensional metallic structures guided by compressive buckling. *Extrem. Mech. Lett.* **11**, 105–110 (2017).
13. Z Yan, J Rogers, Controlled mechanical buckling for origami-inspired construction of 3d microstructures in advanced materials. *Adv. functional materials* **26**, 2629–2639 (2016).
14. I Andrade-Silva, M Adda-Bedia, MA Dias, Foldable cones as a framework for nonrigid origami. *Phys. Rev. E* **100**, 033003 (2019).
15. JA Faber, JP Udani, KS Riley, AR Studart, AF Arrieta, Dome-patterned metamaterial sheets. *Adv. Sci.* **7**, 2001955 (2020).
16. MC Liu, L Domino, ID de Dinechin, M Taffetani, D Vella, Snap-induced morphing: From a single bistable shell to the origin of shape bifurcation in interacting shells. *J. Mech. Phys. Solids* **170**, 105116 (2023).
